# Improved Stability of Rifampicin in the Presence of Gastric-Resistant Isoniazid Microspheres in Acidic Media

**DOI:** 10.3390/pharmaceutics12030234

**Published:** 2020-03-05

**Authors:** Chiluba Mwila, Roderick B. Walker

**Affiliations:** 1 Division of Pharmaceutics, Faculty of Pharmacy, Rhodes University, Grahamstown 6140, South Africa; mwilachiluba@gmail.com; 2 School of Health Sciences, Department of Pharmacy, University of Zambia, Lusaka 10101, Zambia

**Keywords:** isoniazid, rifampicin, gastric-resistant microspheres, solvent evaporation, microporous microspheres, design of experiments, stability, optimization

## Abstract

The degradation of rifampicin (RIF) in an acidic medium to form 3-formyl rifamycin SV, a poorly absorbed compound, is accelerated in the presence of isoniazid, contributing to the poor bioavailability of rifampicin. This manuscript presents a novel approach in which isoniazid is formulated into gastric-resistant sustained-release microspheres and RIF into microporous floating sustained-release microspheres to reduce the potential for interaction between RIF and isoniazid (INH) in an acidic environment. Hydroxypropyl methylcellulose acetate succinate and Eudragit^®^ L100 polymers were used for the manufacture of isoniazid-loaded gastric-resistant sustained-release microspheres using an o/o solvent emulsification evaporation approach. Microporous floating sustained-release microspheres for the delivery of rifampicin in the stomach were manufactured using emulsification and a diffusion/evaporation process. The design of experiments was used to evaluate the impact of input variables on predefined responses or quality attributes of the microspheres. The percent degradation of rifampicin following 12 h dissolution testing in 0.1 M HCl pH 1.2 in the presence of isoniazid gastric-resistant sustained-release microspheres was only 4.44%. These results indicate that the degradation of rifampicin in the presence of isoniazid in acidic media can be reduced by encapsulation of both active pharmaceutical ingredients to ensure release in different segments of the gastrointestinal tract, potentially improving the bioavailability of rifampicin.

## 1. Introduction

**Figure 1:**
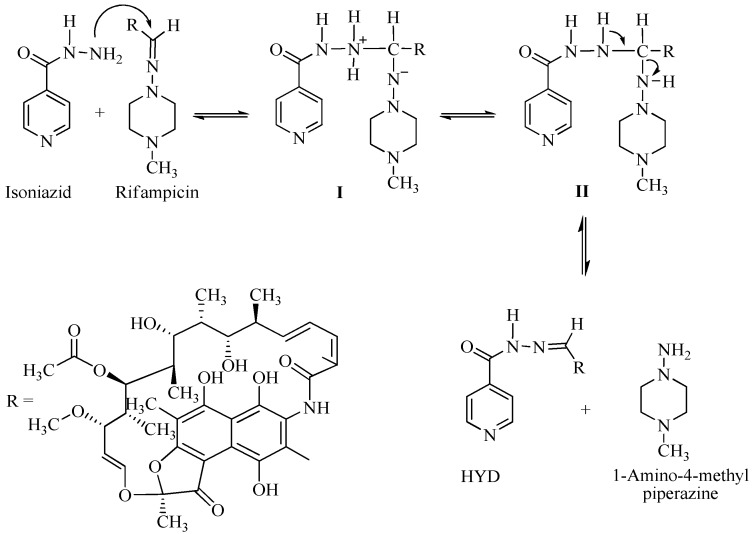
Mechanism of interaction between rifampicin (RIF) and isonicotinylhydrazide (INH), resulting in the formation of hydrazine (HYD).

The greatest concern regarding the use of fixed-dose combinations (FDC) for the treatment of tuberculosis (TB) is the bioavailability of rifampicin (RIF) from FDC anti-TB formulations and medicines [1,2] The decomposition of RIF in the acidic environment of the stomach varies between 8.5% and 50% over the time, corresponding to gastric residence of between 15 and 105 ± 45 min for most dosage units in humans [3,4]. Under acidic conditions in solution, RIF undergoes hydrolysis to yield 3-formyl-rifamycin SV (3-FRSV) and 1-amino 4-methylpiperazine (Figure 1).

The hydrolysis of RIF under acidic conditions is accelerated in the presence of INH [5,6,7]. The production of 3-FRSV, a poorly soluble compound that exhibits in vitro antimicrobial activity and is inactive in vivo, is one of the contributing factors to the poor bioavailability of RIF [8,9]. The accelerated degradation of RIF to form 3-FRSV via reversible binding to the isonicotinyl hydrazone derivative of 3-FRSV with INH under acidic conditions has been postulated to further contribute to the poor bioavailability of RIF when delivered from FDC formulations [4,5,7,10]. RIF in the presence of INH in an FDC has been reported to undergo greater decomposition under acidic conditions of the stomach when compared to RIF when administered via the oral route alone [4]. Therefore less RIF is available for absorption from FDC as compared to RIF administered in a separate formulation, resulting in poor bioavailability of RIF from FDC formulations [3,4,5,7].

Studies suggest that a potential solution to preventing the in situ degradation and low bioavailability of RIF from FDC products lies in the redesign of current FDC products containing RIF and INH so that the two compounds are released in different segments of the GIT. A possible solution is to enteric coat one of the two molecules so that only one molecule is released in the stomach and the other in the ileum [11]. As INH (pKa = 2) is protonated in acidic media, it is unlikely to permeate readily through the stomach mucosa and is primarily absorbed from the small intestine [11]. Therefore, it may be appropriate to formulate INH and RIF for site-specific release in the ileum and stomach, respectively.

Several strategies have been used to prevent the interaction between RIF and INH in the stomach in order to improve the stability of RIF under acidic conditions in an attempt to enhance bioavailability [12,13,14,15,16,17,18,19,20]. This manuscript presents a different approach in which INH was formulated as gastric-resistant sustained-release microspheres with polymers traditionally used for enteric coating, viz., Eudragit^®^ L100 (Rohm Pharma GmbH, Darmstadt, Germany) and hydroxypropyl methylcellulose acetate succinate (HPMC-AS); and RIF was formulated as microporous floating sustained-release microspheres.

## 2. Materials and Methods

### 2.1. Manufacture and Dissolution Testing of Gastric Resistant Sustained Release Isoniazid (INH) Microcapsules

**Figure 2:**
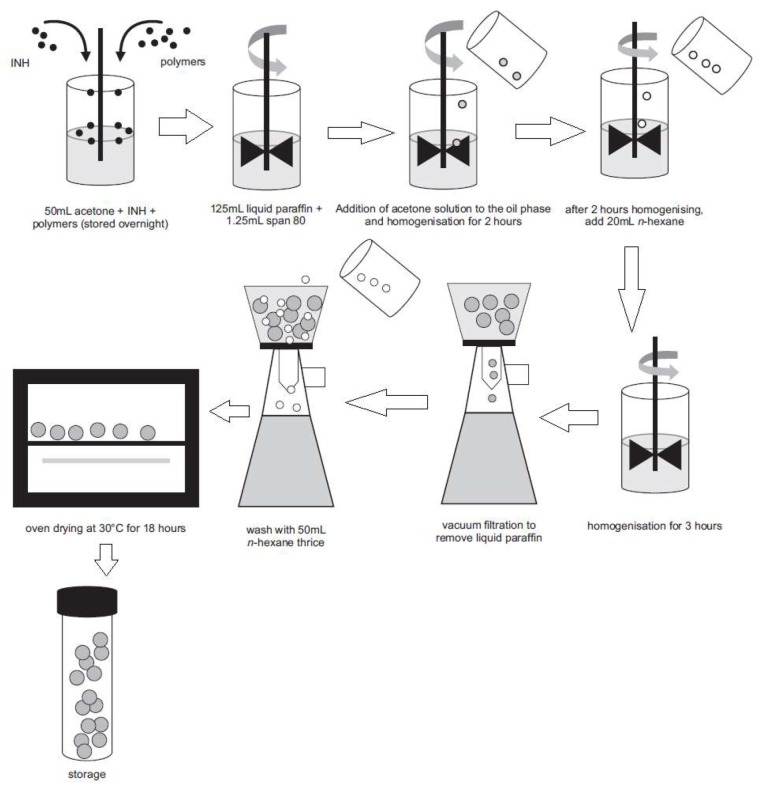
Manufacture of INH microspheres by solvent evaporation. Adapted from Khamanga et al. [21]. Dissolution Technologies, 2009.

Microcapsules were manufactured using a modified emulsification and solvent evaporation approach [21] shown in Figure 2.

In this study, non-aqueous emulsions of acetone (dispersed phase) and liquid paraffin (continuous phase or dispersion medium) were used as these solvents reduce partitioning of the highly water-soluble INH from the microspheres that are formed [21,22,23]. Acetone was selected as the dispersed phase as it is immiscible with liquid paraffin [21,24]. Hydroxypropylmethylcellulose acetate succinate (HPMC-AS) (Shin-Etsu Chemicals Co., Ltd., Tokyo, Japan) was dispersed in 50 mL acetone (Associated Chemical Enterprises, Southdale, South Africa) in a 400 mL beaker and the mixture left to stand overnight to ensure the polymer was completely dissolved, after which Eudragit^®^ L100 (Rohm Pharma, GmbH, Darmstadt, Germany), microcrystalline cellulose (MCC) Avicel^®^ PH101 (FMC, Philadelphia, PA, USA) and an accurately weighed quantity (0.75 g) of INH (China Skyrun Industrial, Tsim Sha Tsui, Hongkong, China) were dispersed in the acetone solution. Light liquid paraffin (ADC Laboratories, Durban, South Africa) (125 mL) containing 1% *v/v* Span 80 (Sigma Aldrich, South Africa) was placed in a 250 mL beaker and agitated with a homogenizer (Virtis Company, New York, NY, USA) fitted with a four-blade ‘‘butterfly’’ propeller of 50 mm diameter to produce an homogeneous oily phase. The entire volume of the previously prepared acetone solution was poured into the oily phase and dispersed. The system was maintained at 22 °C to allow the acetone to evaporate. The amount of liquid paraffin, volume of the acetone solution used, MCC Avicel^®^ PH101 and INH content were kept constant for all batches manufactured. After 2 h of homogenization, 20 mL n-hexane (VWR Chemicals, Fontenay-sous-Bois, France) was added as the non-solvent to harden the microspheres and stirring continued for a further 3 h. The hardened microcapsules were collected using a Buchner funnel and washed 4 times with n-hexane (50 mL) to remove any residual liquid paraffin. The microspheres were dried in an oven maintained at 30 °C for 18 h and then screened to remove any residual powder prior to storage in well-sealed amber bottles (50 mL). The yield of microspheres was calculated using Equation (1).

(1) $$\%\;\mathrm{Yield}=\frac{\mathrm{Mass}\;\mathrm{of}\;\mathrm{microspheres}\;\mathrm{obtained}\;\left(g\right)}{\mathrm{Theoretical}\;\mathrm{mass}\;\mathrm{of}\;\mathrm{microspheres}\;\left(g\right)}\;\times \;100$$

### 2.2. Optimization of INH Microspheres and Evaluation of Model Adequacy

A hybrid statistical experimental design was used to assess the influence of input variables on selected responses. The input variables selected included the amount of HPMC-AS (A), Eudragit^®^ L100 (B), and the speed of homogenization (C). The input variables and their ranges, as well as the responses monitored and analyzed, are summarized in Table 1. The polymer concentration ranges and levels used for the design of experiments were derived from preliminary investigations. Eleven (11) experiments were required for the three input variables to be evaluated using Design^®^ Expert (Version 7.01, Stat-Ease Inc., Minneapolis, MN, USA) statistical software. The results of statistical analysis were then used in the optimization process to select input variables that would yield the required responses.

A number of statistical parameters were assessed to establish the suitability of proposed statistical models for use in formulation optimization. The model F-value is used to ascertain the utility of a model to which the data have been fitted and establish whether the model is the best fit for a set of data. The F-value is a ratio of explained and unexplained variability, and the larger the value for F, the more useful a model [25,26]. The coefficient of variation (% CV) is a ratio of the standard deviation and mean and is indicative of the normalized measure of dispersion of a probability distribution [27,28]. The % CV is a measure of the reproducibility of a model; a value <10% is desirable [29]. Adequate precision compares predicted values located at the design points to the average prediction error. A ratio >4 indicates that adequate model discrimination exists [25,29]. Different coefficients of correlation (R^2^) were used to establish whether a model is able adequately to describe all experimental data under consideration [25,29]. The R^2^ coefficient has a value between 0 and 1, and the closer the value to 1, the more reliable the model. The adequacy of a model was also investigated by examining residuals that are differences between observed and predicted responses [29]. Residuals are examined using normal probability plots of residuals, and a plot of residuals versus predicted responses. If a model is adequate, the points of the normal probability plot of residuals should fall on a straight line, and the location of points on a plot of residuals versus predicted responses should be structureless and not follow a specific pattern [25,26,29]. PRESS is the sum of squares of residuals and is a measure of the discrepancy between experimental data and those estimated by a model. A low value for PRESS indicates a good fit to the model selected [25]. Box-Cox plots of normality are used when transformation was required to increase the applicability and usefulness of an applied statistical test [30].

The polynomial equations in which the dependent and independent variables are related were used in combination with observations made during the manufacturing process to identify and optimize formulation variables that would produce microspheres that met the target responses. The desired target constraints for INH and RIF microspheres were entered into Design^®^ Expert Version 7.01 software (Stat-Ease Inc., Minneapolis, MN, USA) and possible options of combinations of input variables that would yield these outputs were identified. The input variables selected were then used to manufacture three batches of what was considered the optimized option. The experimentally generated outputs measured following evaluation of optimized batches were then compared to the predicted responses generated by Design Expert software to assess the prediction accuracy of the optimization process.

### 2.3. Manufacture and Dissolution Testing of Microporous Floating Sustained Release RIF Microspheres

Microspheres were manufactured using a modified emulsification approach and solvent diffusion/evaporation described in Section 2.1. Eudragit^®^ RLPO (Rohm Pharma, GmbH, Darmstadt, Germany), Ethylcellulose (EC) grade N50 (Hercules Incorporated, Aqualon Division, NC, USA) and MCC Avicel^®^ PH101 were dissolved/dispersed in 50 mL of an organic solvent system comprised of dichloromethane (DCM) (Merck Ltd., Wadeville, Gauteng, South Africa) and acetone in a 3:2 ratio in a 400 mL teflon beaker (Lasec^®^, Cape Town, South Africa). RIF (China Skyrun Industrial, Tsim Sha Tsui, Hongkong, China) and anhydrous d-glucose used as a pore-forming agent (SAARCHEM Ltd., Muldersdrift, Gauteng, South Africa) were mixed in a mortar and approximately 10 mL 95% *v/v* absolute ethanol was added and the powder mixture then levigated to ensure a homogenous paste was produced, prior to air drying at 22 °C for 24 h. A predetermined amount of the RIF and d-glucose mixture containing 1.0 g RIF and desired amount of d-glucose was then added to the polymer solution and left to stand at 22 °C for 1 h, to ensure complete dissolution of the RIF. Light liquid paraffin (125 mL) containing 1% *v/v* Span^®^ 80 was placed into a 250 mL beaker and agitated at 1000 rpm with a Model SS10 overhead stirrer (Stuart^®^-Equipment, Staffordshire, UK) fitted with a four-blade butterfly propeller of 50 mm diameter, for 10 min. The total volume of liquid paraffin was then poured into the teflon beaker containing the polymer and RIF solution and stirred continuously at 1250 rpm for 1.5 h. In order to produce modified RIF microspheres with better floating characteristics, the manufacturing process was modified slightly by accurately weighing sodium bicarbonate (1.0 g) and citric acid (0.5 g) and adding these to the polymer and RIF solution/dispersion that had been prepared as previously reported, vide infra. Hardened microcapsules were collected using a Buchner funnel and washed four (4) times with 50 mL n-hexane to remove any residual liquid paraffin. The microspheres were air-dried in a dark cupboard at 22 °C for 24 h after which the product was sieved to remove residual powder, prior to storage in well-sealed 50 mL amber bottles.

### 2.4. Optimization of RIF Microspheres and Evaluation of Model Adequacy

A Box-Behnken design was used to generate seventeen experiments for which the input variable levels were derived from preliminary studies. The experiments were generated using Design^®^ Expert Version 7.01 (Stat-Ease Inc., Minneapolis, MN, USA) software. The input factors studied were Eudragit^®^ RLPO, EC and anhydrous d-glucose content. The input levels and responses monitored are summarized in Table 2. The evaluation of model adequacy and formulation optimization process was undertaken, as described in Section 2.2.

### 2.5. Differential Scanning Calorimetry

DSC thermograms were generated using a Model DSC 7 (Perkin Elmer^®^, Norwalk, CT, USA), fitted with a PC control unit TAC 7 (Perkin Elmer^®^, Norwalk, CT, USA) at a heating rate of 10 °C/min and a nitrogen flow rate of 20 mL/min. Approximately 3.0 mg of individual samples and 1:1 mixtures of the drugs and polymers to be tested for compatibility, were weighed directly into aluminum DSC pans and hermetically sealed prior heating at a constant rate using an empty pan as the reference. The samples were placed directly onto a micro hot stage DSC and thermograms were generated at temperatures between 30 and 440 °C. The DSC cell was calibrated for temperature and enthalpy with indium (mp. 156.6 °C; ΔH_fus_ = 28.4 j/g) and the resultant data were analyzed using Pyris^TM^ Manager Software (Perkin Elmer^®^, Norwalk, CT, USA).

### 2.6. Fourier Transform Raman Spectroscopy (FTRS)

A Bruker Ram II (Billerica, MA, USA) fitted with a liquid nitrogen germanium detector and CaF_2_ beam splitter was used to record FT Raman spectra. Powder samples of INH and RIF and respective microcapsules were packed separately into small stainless steel cups and subjected to analysis at 1064 nm YAG with a laser beam of 20 mW to generate the Raman scattered light. The FT Raman spectra were recorded over the wavelength range of 50 to 4000 cm^−1^.

### 2.7. Determination of the Particle Size Distribution

The size distribution of the microspheres was determined using a mechanical shaker and sieve stack approach. The nest of sieves was vibrated at 100 rpm for 5 min using a mechanical shaker (Kraemer Ektronic GmbH, Darmstadt, Germany) containing standard sieves with apertures of 315, 800, 1250, and 2000 µm. The weight of the microspheres retained on each sieve was established and the percent mass relative to the cumulative mass retained on each sieve calculated. The mean particle size distribution of the microspheres was determined by measuring the mean microscopic diameters of microspheres (n = 20) using Olympus^®^ AnalySIS Soft Imaging System (Olympus^®^, Munster, Germany).

### 2.8. Bulk and Tapped Density

The tapped bulk density of the microspheres was determined using a model SVM 203 tapped density tester (Erweka GmbH, Heuseastamm, Germany) at a rate of 220 taps per minute for two (2) min. Microspheres were loaded into a 25 mL graduated measuring cylinder and the volume before (bulk volume) and after tapping (tapped volume) determined. Carr’s index (CI; Carr (1965)) [22,31] and Hausner’s ratio (HR; Hausner (1967)) [23,32] were then calculated.

### 2.9. Analytical Method and Chromatographic Conditions

A validated stability-indicating high performance liquid chromatographic (HPLC) method developed and validated for the simultaneous determination of RIF, INH, and pyrazinamide (PZA) was used for analysis. Separation was achieved with gradient elution, and a summary of chromatographic conditions used is listed in Table 3.

The HPLC system consisted of a Waters^®^ Alliance Model 2695 separation module equipped with a solvent delivery module, auto sampler, online degasser and a Model 2998 PDA Detector (Milford, MA, USA) set at 254, 265, and 268 nm for the monitoring of RIF, INH, and PZA, respectively. Data acquisition, processing, and reporting were achieved using Waters^®^ Empower 2 software (Milford, MA, USA). The stationary phase used was a Phenomenex^®^ C_18_ (2), 5 µm, 250 × 4.6 mm i.d. column (Separations, Johannesburg, South Africa). RIF, INH, PZA, and zidovudine, AZT (internal standard) analytical standards were purchased from Sigma Aldrich^®^ (St Louis, MO, USA).

### 2.10. Encapsulation Efficiency

Microspheres containing approximately 50 mg of INH and 100 mg RIF were crushed and dissolved in 50 mL HPLC grade water (100 mL methanol for RIF) and left to stand for 12 h at 22 °C to ensure complete dissolution of the drug. The solution was then vortexed for 5 min (1 h for RIF) and filtered through a 0.45 µm HVLP membrane filter (Millipore Millex-HV, Bedford, MA, USA) prior to further handling. A 1.0 mL aliquot each of INH and RIF solution were diluted to 10 mL with HPLC grade water (methanol for RIF). A 0.6 mL aliquot of the diluted solution was then mixed with 0.3 mL of 300 µg/mL of zidovudine (AZT), the internal standard, in an amber HPLC sample vial, vortexed and then analyzed using the validated reversed-phase HPLC method described in Section 2.9. The percent encapsulation efficiency, % EE, was calculated using Equation (2) [33].

(2) $$\%\;\mathrm{EE}=\frac{\mathrm{Real}\;\mathrm{loaded}\;\mathrm{drug}}{\mathrm{Theoretical}\;\mathrm{loaded}\;\mathrm{drug}}\;\times \;100$$

### 2.11. Scanning Electron Microscopy

The shape and surface morphology of the microspheres were investigated using scanning electron microscopy (Tescan, VEGA LMU, Czech Republic). The microspheres were mounted onto a double-sized carbon stub and placed onto a sample disc carrier (3 mm in height and 10 mm diameter). The sample was sputter-coated with gold under vacuum of 0.25 Torr (Balzers Union Ltd., Balzers, Lichtenstein) prior to imaging at 20 KV with an electron beam.

### 2.12. Assessment of Dissolution and Stability of Rifampicin in the Presence of Isoniazid

In order to assess the stability of RIF in 250 mL 0.1 M HCl (pH 1.2) in the presence of INH, in vitro release studies were conducted using a Vankel Bio-Dis USP Apparatus 3 maintained at 37.0 ± 0.5 °C. The sustained release microporous gastroretentive microspheres (RIF 150 mg) and gastric-resistant microspheres (INH 75 mg) were packed into size 00 gelatin capsules and placed in the inner dissolution cylinder fitted with mesh screen size 177 µm at the top and 74 µm at the bottom to prevent leakage of the dosage form and/or API powder whilst permitting adequate drainage of the dissolution medium [34]. The samples were agitated at 10 dips per minute (dpm), and samples were withdrawn at 0.5, 2, 4, 6, 8, and 12 h. The percent RIF recovered from RIF microspheres alone, commercially available capsules (Rimactane^®^ 150 mg, Norvatis), and RIF API separately, and in the presence of INH tablets (BE-TABS isoniazid 100, Ranbaxy), INH API, and INH microspheres was established using the validated HPLC method reported in Section 2.9. The dissolution testing of commercially available RIF capsules, INH API, and RIF API separately and in the presence of commercially available INH tablets and manufactured INH gastric resistant sustained-release microspheres was undertaken for 2 h, whilst that of the dosage form comprised of manufactured RIF microporous microspheres and INH gastric resistant microspheres was conducted for 12 h.

### 2.13. In Vitro Buoyancy Studies

A 250 mg sample of microspheres was placed into 900 mL pH 1.2 0.1 M HCl in USP Apparatus 2 dissolution vessels maintained at 37.0 ± 0.5 °C. The time taken for the microspheres to move from the bottom of the flask to the surface of the dissolution fluid was monitored and recorded, in seconds, as the floating lag time for the microspheres [35]. The medium was agitated with a paddle at 100 rpm for 12 h after which the microspheres that remained afloat were recovered, separately from those that had settled, and air-dried at 22 °C for 24 h after which the fractions were weighed. The buoyancy was calculated using Equation (3) [36].

(3) $$\%\;\mathrm{Buoyancy}=\frac{\mathrm{Qf}}{\mathrm{Qf}+\mathrm{Qs}}\times 100$$

where,

Qf = weight of floating microspheres, and

Qs = weight of settled microspheres.

## 3. Results and Discussion

### 3.1. Drug-Excipient Compatibility Studies

**Figure 3:**
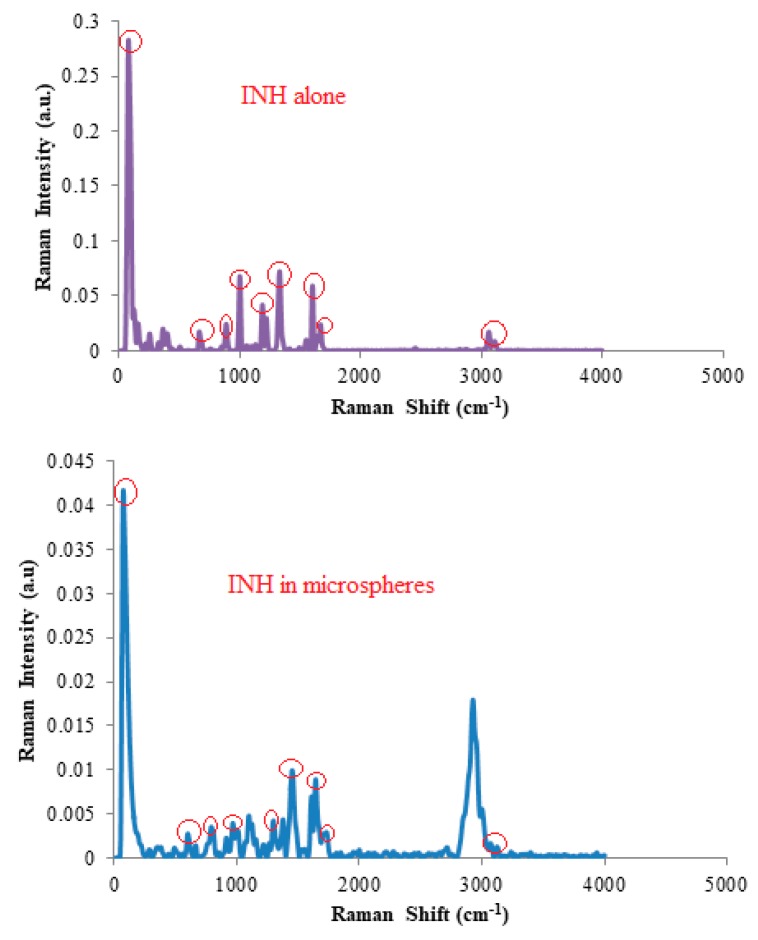
FT Raman spectral data for INH alone and in microspheres.

The thermograms of mixtures of INH and polymers (see Supplementary Materials Data) revealed a minimal decrease in the melting point for INH with associated broadening of the base of peaks, indicating a slight reduction in the crystallinity of INH when in mixtures. The FT Raman frequencies of vibration for the different functional groups for INH powder and microspheres, in relation to what has been reported in literature [37] revealed no significant shifts in frequency for the key functional groups of INH following manufacture as shown in Figure 3, suggesting that significant and/or detrimental interactions between INH and excipients was unlikely.

**Figure 4:**
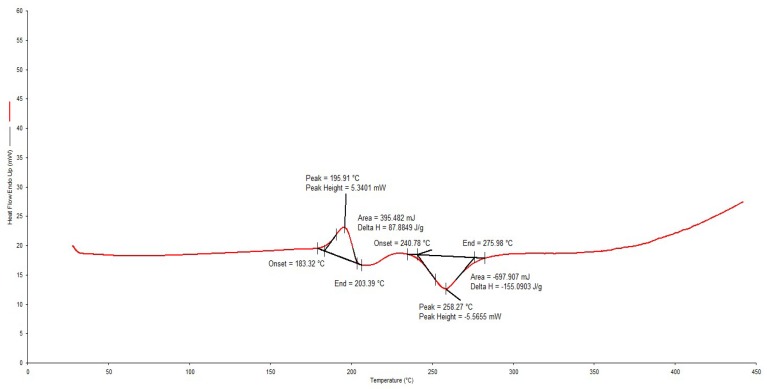
Typical DSC thermogram for RIF generated at a heating rate of 10 °C/min over the temperature range 30–400 °C.

The DSC thermogram for RIF depicted in Figure 4 reveals a melting endotherm between 183.32–203.39 °C, immediately followed by an exothermic recrystallization event, which is characteristic of a solid–liquid–solid transition and finally a decomposition event at 240.78–275.58 °C. These results are similar to previously reported data [38].

The thermogram for the 1:1 mixture of RIF and d-glucose revealed a melting endotherm for RIF between 210.04–231.77 °C, with no recrystallization exothermic event and decomposition between 231.77–279.94 °C. This behavior suggests a more rapid transition from polymorphic form II to form I in the presence of d-glucose and manifests as a decrease in the melting point of RIF in the presence of d-glucose. This phenomenon, however, requires further investigation to determine the biopharmaceutical significance of the transition. Overall, DSC results (Supplementary data) suggested that the intended excipients were compatible with RIF and may be used in the same formulation composition.

The infrared absorption spectra (see Supplementary Materials Data) for 1:1 mixtures did not reveal any significant shifts in the frequency of resonation for RIF, as shown in Table 4.

The minor shifts in the FTIR frequencies of resonance observed for the functional groups of RIF were attributed to potential weak hydrogen bonding, further confirming the absence of potential interactions between RIF and the excipients to be used for the manufacture of the floating microspheres. However, real-time long term stability studies would be necessary to ensure this is, indeed, the case.

### 3.2. Manufacture of Isoniazid Microspheres, Evaluation of Model Adequacy, and Formulation Optimization

Non-aqueous emulsions of acetone (dispersed phase) and liquid paraffin (dispersion medium) were used in this study as these liquids prevent partitioning of highly soluble drugs from microspheres [21,22,23]. Acetone was selected as the dispersed phase due to its poor miscibility with liquid paraffin [21,24].

The actual experiments conducted and the responses generated from the hybrid design are summarized in Table 5.

Analysis of Variance (ANOVA) was applied to the responses to identify input variables that had a significant effect on the responses monitored. The evaluation of model adequacy and ANOVA was conducted for all the monitored responses; however, only details relating to INH release in pH 6.8 0.1 M phosphate buffer at 24 h will be given for illustrative purposes.

**Figure 5:**
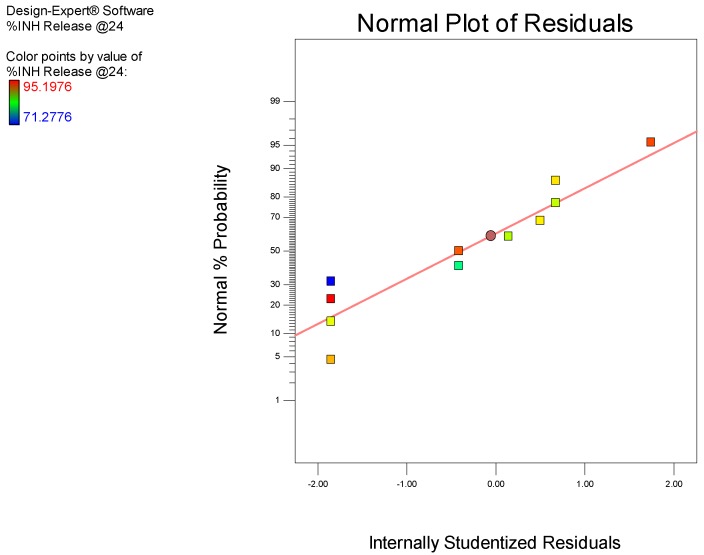
Normal plot of residuals for % INH released in pH 6.8 0.1 M phosphate buffer at 24 h.

A 2-factor interaction (2FI) model was selected for the analysis as all diagnostic statistical parameters viz., a *p*-value <0.05, % CV, R^2^, adequate precision, and normal plot of residuals. A model *p*-value of 0.0427 indicates the significance of the model. This implies that the model is able to describe the fitted data accurately and permit navigation of the design space with only a 0.01% chance of model inaccuracy. The observed value of 3.77 for % CV falls within the recommended limit implying that the model is reproducible over time, which is important for any laboratory analytical procedure. A value of 9.045 for adequate precision was calculated, which is greater than the recommended limit, implies that the model can be used to predict experimental outcomes with acceptable accuracy. The R^2^ coefficients obtained were all >0.5, signifying that the model is reliable and that predicted experimental outcomes will be reasonably close to actual values. Residuals examined using the normal probability plots of residuals depicted in Figure 5 suggest that the model used is adequate.

The ANOVA results for % INH released in pH 6.8, 0.1 M phosphate buffer at 24 h are summarised in Table 6.

A summary of the models describing all responses observed, in addition to the significant factors and mathematical equations describing the responses in relation to the independent variables are listed in Table 7.

The percent yield was affected significantly by the concentration of HPMC-AS and Eudragit^®^ L100 concentration, which was in contrast earlier reported results [39] in which yield was unchanged despite changes in the polymer–drug ratio. This was attributed to the formation of sufficiently strong and robust microspheres that could not revert back to powders during the process of filtration, drying, and sieving. Although not statistically significant, an increase in the homogenization speed, particularly >2000 rpm, resulted in a reduction in the yield. The decrease in percent yield associated with an increase in homogenization speed may be attributed to possible break up of o/o droplets in the emulsion preventing aggregation of smaller microcapsules during the formation stage [40].

The results showed that increasing Eudragit^®^ L100 content resulted in an increase in gastric-resistance. This was attributed to the insoluble nature of Eudragit^®^ L100 in pH 1.2 dissolution fluid [41], which could have prevented the entry of the dissolution medium into the microspheres to dissolve INH. In addition, the increase in the diffusional path length for INH to be released from the core of the microspheres resulted in a delay in release. The concentration of HPMC-AS did not have a significant impact on gastric-resistance, although increasing the polymer concentration resulted in a reduction in the percent INH released in acidic media. The reduction in percent INH released was attributed to low solubility in acid medium and flexibility of the polymer in acidic environments due to the high glass transition temperature of HPMC-AS in the unionized state in acidic media that decreases drug mobility [42]. HMPC-AS is however reported to be approximately 10% ionized at pH < 4, and it is therefore expected to interact with the dissolution medium resulting in the release of any INH located on the surface of the microspheres. Increasing the homogenization speed resulted in a reduction in gastric-resistance and was attributed to the formation of smaller microspheres, which presented a larger surface area for INH release.

**Figure 6:**
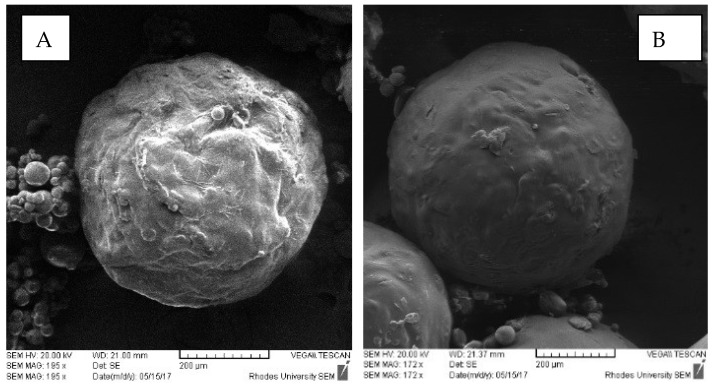
SEM images of INH-loaded microcapsules (**A**) batch INH-006 containing 3.00 g HPMC-AS and 1.00 g Eudragit^®^ L100 and (**B**) batch INH-007 containing 1.00 g HPMC-AS and 3.00 g Eudragit^®^ L100.

Since INH is highly water-soluble, the difficulty in controlling release from microspheres in aqueous media was anticipated. It was expected that a formulation using a combination of Eudragit^®^ L100 and HPMC-AS would show significant gastric-resistance. Analysis of SEM images of microspheres (Figure 6) showed that the microspheres had smooth surfaces.

Microspheres in which higher concentrations of HPMC-AS than Eudragit^®^ L100 were used had rough surfaces, which were attributed to possible deposition of INH particles on the surface of the microspheres. In general, the results suggest significant promise for use in preventing the release of INH in an acidic medium and that high gastric resistance may be achieved when the Eudragit^®^ L100 content is high.

The results show that the release of INH was affected by HPMC-AS content with increasing polymer content resulting in a decrease in the % INH released, which was attributed to an increase in the thickness of the diffusional barrier for INH. The interactive effects of HPMC-AS and Eudragit^®^ L100 content had a significant impact on INH release that was confirmed by a *p*-value of 0.0310 for the model term AB, suggesting that the ratio of HPMC-AS to Eudragit^®^ L100 was an important factor impacting the release of INH. The results show that increasing the homogenization speed resulted in an increase in INH release that could be attributed to the production of small microcapsules with a narrow size distribution, resulting in a large surface area and a reduced barrier for INH diffusion. An increase in the Eudragit^®^ L100 reduced INH release, possibly due to an increase in the thickness of the diffusion pathway. In general, the results suggest that INH release was retarded and sustained despite the use of polymers, which were expected to dissolve in 0.1 M phosphate buffer pH 6.8, and this was attributed to the presence of HPMC-AS in the formulation. The significant impact of HPMC-AS content on INH release can be attributed to the physicochemical characteristics of this polymer. The succinate functional groups of HPMC-AS have a pKa of approximately 5; therefore, the polymer is <10% ionized at pH values <4 and is at least 50% ionized at pH values ≤ 5 [42]. Due to the presence of relatively hydrophobic methoxy and acetate substituents, HPMC-AS is water-insoluble when unionized and remains sparingly soluble and predominantly as colloidal polymer aggregates at intestinal aqueous solution pH, viz., pH 6.0–7.5 [42]. The results reveal that >80% INH would be released within 24 h, except from microspheres of batch INH004 that were manufactured with the highest total polymer content.

Increasing the amount of polymer used resulted in an increase in the % EE due to an increase in the amount of polymer available for encapsulation of RIF and similar data has been reported [43,44,45,46]. The contribution of a high polymer content to the % EE can be interpreted in two ways, viz., (i) in highly concentrated solutions, the polymer precipitates more rapidly onto the surfaces of the dispersed phase and prevents API diffusion across the phase boundary [47] or (ii), the use of a high concentration of polymer results in an increase in the viscosity of the solution and delays API diffusion within the polymer droplets [48].

Increasing the homogenization speed reduced the % EE regardless of the total polymer content used. The decrease in % EE associated with an increase in homogenization speed may be attributed to the break-up and disruption of small o/o emulsion droplets, thereby preventing encapsulation of INH, as the aggregation of smaller microspheres during the formation stage could not be achieved [49]. In general, the % EE for INH from formulations was relatively high (89.7% highest), justifying the selection of the o/o emulsification method of manufacture of microspheres with the water-soluble INH as the payload.

The desired target constraints for the highest % yield, minimal % INH released at 4 h in 0.1 M HCl, >80 % INH released at 24 h in pH 6.8 buffer and the highest encapsulation efficiency possible were set in Design^®^ Expert software and possible combinations of input variables that would yield these outputs identified. The input variables selected were then used to manufacture three batches, viz., INH-012, INH-013, and INH-014, that were considered the optimized option. The experimentally generated output data following the evaluation of the three optimized batches were then compared to the predicted responses generated by Design Expert software (Table 8) to assess the prediction accuracy of the optimization process.

**Figure 7:**
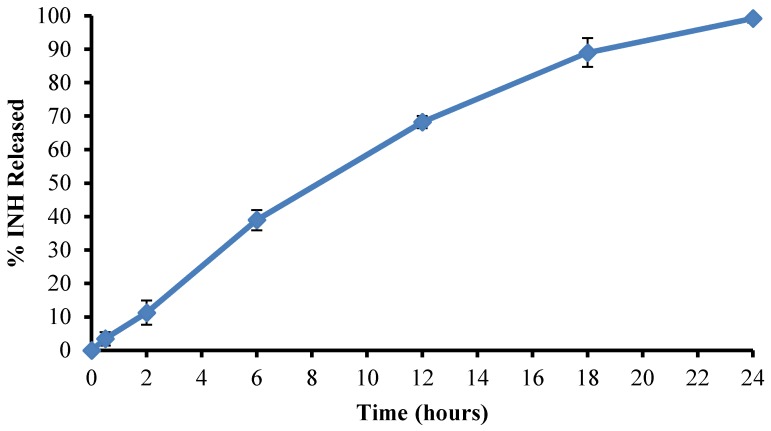
Mean dissolution profile (n = 6) of % INH released from the optimized formulation in pH 6.8 0.1 M phosphate buffer.

The prediction accuracy of the process was established by calculating the residuals (R) and percent prediction error (% P.E.) for the three optimized batches. The residuals and % P.E. for the responses were considered acceptable for % yield, encapsulation efficiency, and % INH released at 24 h in pH 6.8 buffer where the % P.E. was <12%. The % P.E. values for % INH released at 4 h in 0.1 M HCl and % INH released at 12 h in pH 6.8 buffer were very high, indicating the models used were poor predictors for these two responses suggesting that the use of a hybrid experimental design may not be accurate and perhaps the absence of replication points and a lack of fit of parameter for model evaluation could be a disadvantage and an alternate experimental design should be considered. The need to select models that have parameters with a good fit should also be prioritized. The use of these input variables produced an average yield of 52.41 ± 1.80% with significant gastric-resistant microspheres as only 0.523 ± 0.13% INH was released after 2 h and 8.68 ± 0.16% released after 4 h in 0.1 M HCl. The optimized formulation exhibited an average encapsulation efficiency of 82.92 ± 3.80%. The dissolution profile for % INH released from the optimized formulation in pH 6.8 0.1 M phosphate buffer is depicted in Figure 7.

### 3.3. Manufacture of RIF Microspheres, Evaluation of Model Adequacy, and Formulation Optimization

**Figure 8:**
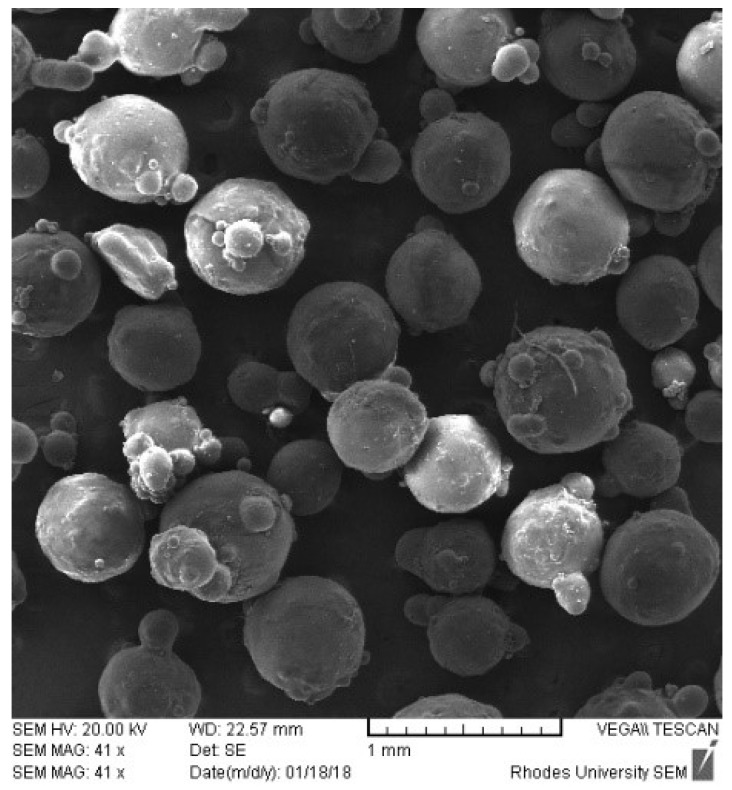
SEM image of RIF microspheres.

The use of a single phase of acetone resulted in the production of non-spherical microparticles with a poor yield that was attributed to the failure to form stable emulsion droplets of the polymer solution in liquid paraffin [50,51]. DCM is nonpolar, is miscible in oil, and was included to decrease the polarity of acetone to enhance the formation of a stable emulsion [50,52]. The use of DCM alone resulted in the formation of large droplets that aggregated and the use of a solvent mixture of DCM and acetone was considered suitable for this process. Acetone and DCM are miscible [53], and the use of DCM and acetone in a 3:2 ratio produced smooth spherical particles (Figure 8) that did not adhere to other particles and the walls of the beaker, and this ratio was selected for the manufacture of floating RIF particles. Following the addition of the RIF solution to liquid paraffin, small discrete oil droplets stabilized with Span^®^ 80 were formed.

The responses generated from Box Behnken Design (BBD) experiments (Supplementary Materials Data) were subjected to ANOVA to identify input variables that had a significant effect on the responses monitored. The evaluation of model adequacy and ANOVA was conducted for all responses, as described in Section 3.2; however, only details relating to RIF release at 0.5 h in 0.1 M HCl are provided for illustrative purposes.

A summary of ANOVA results for the % RIF released at 0.5 h is listed in Table 9.

A quadratic model was selected for this analysis as all diagnostic parameters, viz., *p*-value, PRESS, R^2^, adequate precision, and diagnostic plot of the normal plot of residuals implied that the model was adequate for use. Model terms with values of Prob > F < 0.0500 indicate that the model terms are significant.

A summary of the models describing all observed responses, in addition to the significant factors and mathematical equations describing the responses in relation to the independent variables are listed in Table 10.

The results revealed that increasing the amount of Eudragit^®^ RLPO and EC resulted in an increase in the % yield and was attributed to the increase in total polymer available for microencapsulation. These results were in contrast to those reported elsewhere [39] in which yield was unchanged despite changes in the API–polymer ratio. It was observed that an increase in pore-forming agent d-glucose, whilst keeping Eudragit^®^ RLPO at the lowest content, resulted in the formation of microspheres that may break, and the reduction in yield could be as a result of damage of microspheres during filtration, drying and sieving and has been reported [54]. An increase in the Eudragit^®^ RLPO content whilst keeping d-glucose content low resulted in an increase in % yield and was attributed to the formation of microspheres with a resilient microsphere wall.

An increase in the Eudragit^®^ RLPO and EC content resulted in a decrease in the % RIF released with the highest % RIF released when these two polymers were used at the lowest levels. This effect was attributed to an increase in the thickness of the barrier coat, thereby increasing the diffusional path length through which RIF must diffuse to be released [55,56]. An increase in the amount of EC used resulted in less dissolution medium entering the microspheres and reducing the release of RIF through a reduction in dissolution of d-glucose forming fewer channels through which RIF diffusion could occur and erosion of the microsphere matrix [57,58]. The results also revealed that maintaining EC at low levels and increasing the Eudragit^®^ RLPO content resulted in an increase in % RIF released and these results were similar to previously reported data [59] in which this was attributed to the rapid ingress of dissolution medium into the microspheres due to the presence of quaternary ammonium ions in Eudragit^®^ RLPO that facilitate dissolution of RIF present on the surface of microspheres, as well as promoting outward diffusion of RIF from the core. Increasing d-glucose content resulted in an increase in the % RIF released while increasing the amount of Eudragi^t®^ RLPO decreased the % RIF released with the highest % RIF released when d-glucose content was at the highest and Eudragit^®^ RLPO content at the lowest levels. As RIF is hydrophobic, release from the microspheres would be dependent on the availability of pores and/or erosion or dissolution of the dosage form [59]. The dissolution of d-glucose is expected to create pores in the surface(s) of the microsphere coating, facilitating the entry of dissolution medium into the microspheres and outward diffusion of RIF from the core of the particle and similar results have been reported [60,61]. It was expected that increasing the amount of d-glucose would result in a greater number of pores forming on the surface(s) of the microspheres with a subsequent increase in the % RIF released due to channel formation, through which diffusion could occur, as has been reported [62,63]. The results revealed that the % RIF released from microspheres for all BBD formulation except batches RIF015 and RIF016 did not exhibit a burst release or dose dumping, a phenomenon that is desirable for sustained release dosage form performance [56]. Since EC acts as a barrier to water [64], it was postulated that the amount of the RIF released in the initial phase was a consequence of dissolution of RIF located at the surface of the microspheres and outward diffusion of RIF through pores created by the permeability imparted on the surface of the microspheres by quaternary ammonium ions present in Eudragit^®^ RLPO that facilitate diffusion of the entrapped RIF from the surface of the microspheres in the initial phase of the dissolution process. In addition, the dissolution of d-glucose may result in the dissolution of RIF particularly on the surface(s) of the microspheres due to improved wettability of the microspheres.

API release rates are reported to be drastically reduced with the increase in the molecular weight of EC used which results in the formation of a stronger film with high tensile strength and elasticity that may resist hydrostatic pressure, ensuring less structural damage to the film due to stress fractures or channel formation [65]. These results were similar to those reported where formulations coated with lower molecular weight EC exhibited faster release rates and extent of API release when compared to formulations coated with higher molecular weight EC [64]. The expected mode of RIF release would be diffusion control and the channels for diffusion would have been created by the dissolution of d-glucose and the number of the channels was expected to increase with an increase in the amount of d-glucose included [60,61]. Eudragit^®^ RLPO was expected to undergo time-dependent erosion and its effect on RIF release may have been a result of the restricted entry of dissolution medium by EC into the microspheres [41] or initial swelling in aqueous media [66] that may impede RIF diffusion. The data for % RIF released at 8 h indicate that the release of RIF would be sustained over 8 h, as approximately 80 % RIF was released from the microspheres for most batches.

The results revealed that increasing the amount of polymer used resulted in an increase in the % EE due to an increase in the amount of polymer available for encapsulation of RIF and similar data has been reported [43,44,45,46].

The rapid formation of microspheres was evident from the short manufacturing times observed, suggesting rapid diffusion of DCM into the continuous phase occurred as it is miscible with liquid paraffin [45]. The diffusion of DCM from the droplets into the continuous phase resulted in precipitation and solidification of polymers and entrapment of RIF. The rapid entrapment of RIF prevented the diffusion of RIF into the continuous phase and generally resulted in high % EE. The results were similar to those reported in which an increase in EE was associated with the use of a co-solvent, miscible with the continuous phase [23]. The results indicate that an increase in the amount of d-glucose used resulted in a reduction in the % EE of RIF that was attributed to possible disruption of the structure of the microsphere wall, and leaving spaces through which diffusion of RIF into the continuous medium could have occurred. Similar results, in which the use of additives insoluble in the dispersed phase was associated with reduced % EE, have been reported [67]. Assessment of the impact of EC content on % EE also revealed that an increase an increase in EC content resulted in an increase in % EE which was attributed to the ability of EC to form strong membrane films with no significant channels for RIF to diffuse through, as stated earlier [59,67,68,69].

The results revealed that increasing the amount of Eudragit^®^ RLPO used, resulted in a reduction in the floating capacity of the microspheres, which was attributed to the permeability of Eudragit^®^ RLPO, due to the quaternary ammonium ion content, to water, thereby increasing the density of the microspheres that has also been reported elsewhere [70]. It has been reported that microspheres manufactured using Eudragit^®^ EPO exhibit low buoyancy that was attributed to the solubility of the polymer in gastric fluids [70]. Increasing the amount of EC used, resulted in an increase in the % buoyancy, in vitro, that was attributed to excellent barrier characteristics to water, thereby preventing entry of dissolution fluid into the microspheres, a consequence of which is that an increase in density of the microspheres is avoided and has been reported [71,72]. Inspection of the relationship between input variables and % buoyancy revealed that an increase in d-glucose content resulted in an increase in buoyancy. An increase in the buoyancy of microspheres with an increase in the content of a pore-forming agent has been reported [73] and was attributed to an increase in the number of pores in the microsphere. The pores created allow entry of dissolution medium that erodes the inner surface of the matrix, resulting in a reduced density of microspheres, thereby making them float. It was anticipated that the creation of pores in the microspheres would result in high buoyancy due to increased erosion resulting in a reduction of the density of the microspheres [73,74]; however, this was not realized as the pronounced effects of Eudragit^®^ on permeability and EC on the escape of imbibed dissolution fluid prevail. This observation pointed to a possibility of the microspheres maintaining their integral architecture, albeit with a change in size and morphology, during dissolution testing. In general, the buoyancy of microspheres of all batches was low, with the highest buoyancy recorded of 68%, suggesting that a significant number of microspheres did not float in acidic solution. Consequently, many of the microspheres would be emptied into the small intestine fairly rapidly in vivo, which has bioavailability implications as the solubility and absorption of RIF are low from this part of the GIT. Therefore, the inclusion of sodium bicarbonate (Aspen^®^ Pharmacare, Port Elizabeth, Eastern Cape, South Africa) to the formulation was considered as CO_2_ is generated when the compound is exposed to acid, which would, therefore, improve the floating and buoyancy of the microspheres [75].

The results revealed that increasing the Eudragit^®^ RLPO content resulted in an increase in floating lag time, with the highest floating lag time recorded when Eudragit^®^ RLPO content was at the highest and EC content at the lowest levels, possibly due to an increase in the density of microspheres following rapid uptake of dissolution fluid by the quaternary ammonium ions present in Eudragit^®^ RLPO [70]. Increasing the amount of EC used resulted in a decrease in floating lag time, possibly due to the barrier properties of EC, which resulted in minimal uptake of dissolution fluid [59]. The results reveal that an increase in d-glucose content resulted in an increase in floating lag time that was attributed to the ability of d-glucose to adsorb dissolution fluid, resulting in an increase in the density of the microspheres during the initial stages of testing. In addition, an increase in the amount of Eudragit^®^ RLPO used also resulted in an increase in the floating lag time due to the ingress of the dissolution medium as a result of quaternary ammonium ions as stated earlier [70]. These results revealed that floating lag time of the microspheres for BBD batches was long and ranged between 110 to 220 s for most formulations. Although some microspheres would float from the bottom of the dissolution vessel to the surface of the dissolution fluid, some sank to the bottom after a short period of time, suggesting these microspheres would be emptied from the stomach rapidly, due to their proximity to the gastric–duodenal junction and that the RIF would not be available for absorption from the stomach. This observation further supports the decision to add a CO_2_ generating component to the formulation.

The modified microspheres exhibited a % buoyancy of 87.66 ± 1.28% and a floating lag time of 15 ± 3.2 s. Furthermore, the microspheres remained buoyant for up to 12 h. The improvement in floating behavior of the microspheres was attributed to the generation of CO_2_ on contact with the dissolution fluid, resulting in the microspheres floating and these observations are similar to what has been reported [76]. The dissolution of sodium bicarbonate and production of CO_2_ may have created spaces within the microspheres into which dissolution medium diffused leading to erosion and reduced density of core [75]. The manufacturing process produced discrete spherical microspheres with a % yield of 97.56%. The % EE of RIF for the modified formulation was 88.26 ± 1.25% that was reduced when compared to that of the optimized formulation, which was 96.99 ± 2.19%. This reduction was attributed to possible disruption of the film structure as a result of the incorporation of sodium bicarbonate and citric acid, which could have left voids through the microsphere core through which RIF may have diffused to reach the continuous medium and these data are similar to what has been reported elsewhere [67].

**Figure 9:**
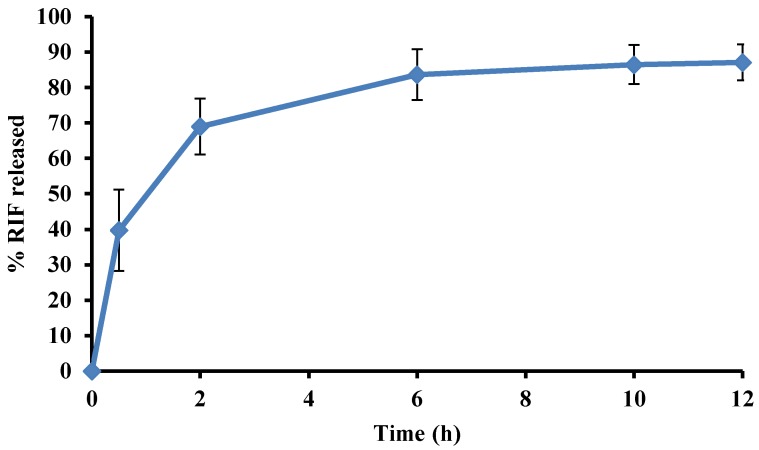
Dissolution profile depicting % RIF released from the modified formulation in pH 1.2 0.1 M HCl after 12 h.

RIF release from the modified microspheres occurred at a faster rate than from the optimized batch. The dissolution profile for % RIF released from modified batch formulation in pH 1.2, 0.1 M HCl is depicted in Figure 9.

The more rapid entry of dissolution medium into the microspheres resulted in a more rapid dissolution of d-glucose, further increasing the channels for diffusion of RIF, as well as increased erosion of the matrix, and these results are similar to those reported elsewhere [54].

The prediction accuracy for this process is summarised in Table 11, and the % PE was in general <10%, with only buoyancy exceeding this limit for two batches. These data suggest that the models used are useful and successfully applied to the development and manufacture of RIF microspheres.

The application of the variables recommended by the optimization process resulted in the production of batches with an average yield of 85.27% ± 3.06%. The release of RIF was sustained over 12 h with 88.82% ± 2.95% RIF released at 12 h. The microspheres exhibited a % buoyancy of 87.66% ± 1.28% (n = 6) and floating lag time of 15 ± 3.2 (n = 6) seconds. The % EE of RIF for the modified formulation was 88.26% ± 1.25%.

### 3.4. Particle Size Distribution

Large INH microspheres were produced at higher total polymer concentrations and lower homogenization speeds. This was attributed to the production of a viscous solution that, when poured into the aqueous phase, resulted in the formation of large droplets leading to the formation of large microspheres [77]. The decrease in particle size associated with an increase in homogenization speed is a consequence of the formation of smaller droplets and the formation of smaller microspheres [30]. The particle size distribution for different batches of microspheres is listed in Table 12.

The intra-batch mean particle size distribution of INH microspheres from the hybrid design batches and that of the optimized batch formulation ranged between 415.76 ± 76.93 to 903.35 ± 197.10 µm.

Most of the RIF microspheres, >90% of the experimental design products in addition to the optimized batch (RIF018) had diameters < 800 µm as summarized in Table 13, and suggested a fairly uniform particle size distribution.

The inter-batch uniform size distribution can be attributed to the use of a constant homogenization speed for all batches, as homogenization is reported to be a key determinant of microsphere size [78]. The intra-batch mean particle size distribution of RIF microspheres from the BBD batches and that of the modified batch formulation ranged between 423.19 ± 121.86 to 620.07 ± 102.67 µm in diameter. Analysis of the intra-batch mean particle size distribution of RIF microspheres revealed that the size of microspheres was wide with a % RSD ranging between 16.56% to 40.72%.

Since the size of microspheres influences the rate of drug release [79], the data suggest that the rate of RIF release from the different batches would be different which, in turn, may result in variable plasma concentration. The % RSD for RIF release at different time points for the modified optimized microspheres ranged between 5.52% to 11.41%, which was within the acceptable range of not >20% for early time points and not >10% at the later time points [80]. The average particle size of 696.22 ± 124.90 µm and particle size distribution of 415.76 ± 76.93 to 903.35 ± 197.10 µm for INH microspheres and the average particle size of 526.49 ± 91.88 µm and particle size distribution of 423.19 ± 121.86 to 620.07 ± 102.67 µm for RIF microspheres are suitable for packing in a size 00 gelatin capsule.

### 3.5. Flowability—Carr’s Index (CI) and Hausner Ratio (HR)

The microspheres exhibited excellent flow characteristics due to their high sphericity. The CI of the microspheres was 4.888 ± 0.5659% and 1.998 ± 0.043% and the HR was 1.058 ± 0.012 and 1.020 ± 0.001 for INH and RIF, respectively, indicating the potential of packability into capsule shells. The INH microspheres were hard and bouncy, which was attributed to the high glass transition temperature of HPMC-AS [42]. The evaporation of acetone results in the conversion of HPMC-AS into a unionized state and the formation of a hard film.

### 3.6. Stability of RIF in Presence of INH

The percent degradation of RIF after 2 h dissolution testing in pH 1.2 0.1 M HCl of Rimactane^®^ 150 capsules, Rimactane^®^ 150 capsules in presence BE-TABS isoniazid 100, RIF API, RIF API in presence of INH API, Rimactane^®^ 150 capsules, RIF API in the presence of INH gastric resistant microspheres, and RIF microporous floating sustained-release microspheres in the presence of INH gastric resistant microspheres are summarized in Table 14.

The results reveal that RIF in Rimactane^®^ 150 capsules degraded with 24.1% lost when tested in the presence of BE-TABS isoniazid 100 and in the presence of INH API, where 24.4% degradation was observed. However, the degradation of RIF from Rimactane^®^ 150 and API was significantly reduced in the presence of INH microspheres, with only 4.05% and 3.86% degradation for Rimactane^®^ 150 and RIF API, respectively. The degradation of RIF after 12 h dissolution in pH 1.2 0.1 M HCl from the microporous floating microspheres in the presence of INH gastric-resistant sustained-release microspheres exhibited degradation of only 4.44%. These results are an improvement in the reported data in which degradation of RIF in the presence of INH was decreased by 11% [16], 10% [81], 11.5% [13], and 12% [19] when different developed technologies were used. These results are similar to those reported in which a 3.6% to 4.8% reduction in RIF degradation, when in the presence of INH, was observed [14]. These results suggest that the degradation of RIF in the presence of INH in an acidic medium can be overcome by encapsulation of both APIs in a manner that permits the product to release each compound in different segments of the GIT.

## 4. Conclusions

The drug delivery technology developed and reported in this study has the potential to ensure that more RIF is available for absorption from the stomach where the solubility and absorption potential for the molecule is high [82] and would likely improve the bioavailability of RIF when delivered with INH, thereby enhancing the clinical response and reducing the likelihood of resistance to RIF developing. However, in vivo studies will be required to establish that this is indeed the case. Furthermore, this approach permits dose titration based on weight for patients requiring different doses of each compound, which also may be of use in reducing the side-effects associated when using long term treatment with existing products. Further studies should be conducted to determine the amount of residual solvent in the microspheres.

## Figures and Tables

**Table 1:** Coded levels for input variables and responses used for the hybrid experimental design.

Factor	Code	Coded Level				
**Independent/Input**						
**Level**		−1.4142	−1	0	+1	+1.4142
HMPC-AS (g)	A	0.585	1.000	2.000	3.000	3.414
Eudragit^®^L100 (g)	B	0.585	1.000	2.000	3.000	3.414
Homogenization Speed (rpm × 100)	C	0.5	1.0	2.0	3.0	3.4
**Dependent/Output**		**Constraint**				
Y_1_ = Percent yield		Maximize				
Y_2_ = % INH released in 0.1 M HCl at 4 h		Minimize				
Y_3_ = % INH released in buffer pH 6.8 at 12 h		Maximize				
Y_4_ = Encapsulation efficiency		Maximize				
Y_5_ = % INH released in buffer pH 6.8 at 24 h		Maximize				

**Table 2:** Input variables (coded) and responses monitored for the Box-Behnken Design.

Factor	Code	Coded Level		
**Independent**				
**Level**		[−1]	[0]	[+1]
Eudragit^®^ RLPO content (g)	A	1	2	3
Ethylcellulose content (g)	B	1	1.5	2
Anhydrous d-glucose content (g)	C	0.25	0.5	0.75
**Dependent**	**Constraints**			
% RIF released at 0.5 h = R_1_	R_1_ ≤ 15%			
% RIF released at 2 h = R_2_	R_2_ = maximize			
% RIF released at 4 h = R_3_	R_3_ = maximize			
% RIF released at 6 h = R_4_	R_4_ = maximize			
% RIF released at 8 h = R_5_	R_5_ = maximize			
Encapsulation efficiency (%) = R_6_	R_6_ = maximize			
Buoyancy = R_7_	R_7_ = maximize			
Lag time (seconds) = R_8_	R_8_ = minimize			
% RIF released at 12h = R_9_	R_9_ = maximize			
% Yield = R_10_	R_10_ = maximize			

**Table 3:** Summary of chromatographic and system suitability parameters.

**Parameter**	**Setting**		
Chromatographic parameters:	-		
Flow rate (mL/min)	1.0		
Buffer pH	3.0		
Starting % MeOH	3.0		
Ending % MeOH	75		
Curve profile	6		
Gradient duration (min)	4		
Injection volume (µL)	10		
Column temperature (°C)	22		
Wavelength (nm)	254 (RIF), 265 (INH), 268 (PZA)		
Mobile phase composition	Methanol (3% *v/v*) and 10 mM phosphate buffer (97% *v/v*)		
**System Suitability Parameters**	**INH**	**PZA**	**RIF**
Theoretical number of plates (N)	8262.616	10,993.81	83,450.80
Capacity factor	1.06560	4.316160	7.77262
Resolution factor	-	21.54936	8.09081
Tailing factor	1.08756	1.099920	1.65720
Retention time	3.261	7.630	12.793
Correlation coefficient (R^2^)	0.9991	0.9995	0.9990
Limit of Detection (LOD) n = 5	0.3 ± 0.17	0.3 ± 1.87	5.0 ± 0.75
Limit of quantitation (LOQ)	0.1	0.1	0.5

**Table 4:** FTIR wavenumber number assignments for RIF and 1:1 mixtures with excipients.

Functional Group	RIF	1:1 Mixture of RIF and				
		Ethylcellulose	Eudragit^®^ RLPO	d-Glucose	Sodium Bicarbonate	Citric Acid
–CH_3_ stretching	2941.77	2936.70	2936.70	2941.77	2941.77	2941.78
–CH_3_O asymmetric stretching	2875.94	2875.94	2875.94	2881.01	2875.94	2875.94
–C=O acetyl stretching	1735.99	1735.99	1735.99	1735.99	1735.35	1735.99
–C=N asymmetric bending	1673.87	1676.40	1676.40	1676.40	1676.40	1673.87
–C=C stretching	1562.64	1560.11	1560.11	1560.11	1562.40	1562.64
–C–N– stretching	1427.81	1426.10	1426.12	1426.12	1426.12	1427.44

**Table 5:** Actual experiments conducted and responses observed using the hybrid Design of Experiments (DOE).

Run	HPMC-AS (g)	Eudragit^®^L100 (g)	Homogenisation Speed (rpm)	Yield (%)	INH Released at 4 h (%)	INH Released at 12 h (%)	Encapsulation Efficiency (%)	INH Released at 24 h (%)
1	3.41	2.00	1500	51.32	17.45	45.35	89.67	80.26
2	2.00	2.00	2000	43.30	29.74	64.44	72.57	89.56
3	2.00	2.00	500	56.52	23.30	55.35	77.42	87.45
4	3.00	3.00	2500	48.32	19.05	36.25	65.16	71.28
5	0.485	2.00	1500	21.91	38.42	65.41	50.51	93.16
6	3.00	1.00	2500	45.83	39.33	53.99	70.99	88.67
7	1.00	3.00	2500	40.00	13.02	59.45	68.65	95.20
8	2.00	0.485	1500	21.14	71.04	55.78	55.48	89.81
9	2.00	2.00	3500	31.83	36.66	68.89	60.84	93.58
10	1.00	1.00	2500	22.27	45.86	67.51	66.72	90.96
11	2.00	3.41	1500	47.17	8.82	47.75	89.41	87.87

**Table 6:** ANOVA data for a 2FI model (partial sum of squares—Type III) for % INH released in pH 6.8, 0.1 M phosphate buffer at 24 h.

Source	Sum of Squares	Df	Mean Square	F-Value	*p*-Value	Prob > F
**Panel A**						
Model	420.28	6	70.05	6.37	0.0427	Significant
A-HPMC-AS	246.99	1	246.99	22.48	0.009	Significant
B-Eudragit^®^ L100	31.57	1	31.57	2.87	0.1654	-
C-Homogenisation speed	3.29	1	3.29	0.30	0.6134	-
AB	116.98	1	116.98	10.65	0.0310	Significant
AC	7.93	1	7.93	0.72	0.4434	-
BC	13.53	1	13.53	1.23	0.3294	-
Residual	43.96	4	10.99	-	-	-
Cor total	464.24	10	-	-	-	-
**Panel B**						
Std. deviation	3.31	-	-	-	-	-
Mean	87.98	-	-	-	-	-
% C.V	3.77	-	-	-	-	-
Press	1655.17	-	-	-	-	-
R^2^	0.9053	-	-	-	-	-
Adjusted R^2^	0.7633	-	-	-	-	-
Adeq precision	9.045	-	-	-	-	-

**Table 7:** Summary of best fit models, significant factors, and equations describing the relationship between input variables and response for INH microspheres.

Response	Fitting Model	Significant Factors	Equation
%Yield-Y_1_	Linear	A-HPMC-AS B-Eudragit^®^ L100	Y_1_ = 39.06 + 9.18A + 7.13B − 3.05C
% INH released at 4 h in pH 1.2 0.1M HCl-Y_2_	Linear	B-Eudragit^®^ L100	Y_2_ = 31.15 − 3.77A − 17.64B + 0.73C
%INH released at 24 h in pH 6.8 0.1 M phosphate buffer-Y_3_	2FI	A-HPMC-AS B-Eudragit^®^ L100	Y_3_ = 87.98 − 5.56A − 1.99B + 0.64C − 5.41AB − 1.41AC − 1.84BC
Encapsulation efficiency (%EE)-Y_4_	2FI	A-HPMC-AS B-Eudragit^®^ L100 C-Homogenisation speed	Y_4_ = 72.49 + 5.77A + 6.76B − 1.48C − 9.44AB − 11.42AC − 7.40BC

**Table 8:** Comparison of predicted and experimentally derived responses.

Batch	Response	Predicted Value	Observed Value	Residual	% P.E.
INH-012	% Yield	51.35	50.41	0.940	1.830
	% INH released at 4 h in pH1.2	5.745	8.510	5.989	48.13
	Encapsulation efficiency (%)	90.81	87.37	3.440	3.788
	% INH released at 24 h in pH 6.8	86.62	90.96	4.340	5.010
INH-013	% Yield	51.35	52.89	1.540	2.999
	% INH released at 4 h in pH 1.2	5.745	8.810	3.219	53.35
	Encapsulation efficiency (%)	90.81	80.79	10.02	11.03
	% INH released at 24 h in pH 6.8	86.62	99.20	12.58	14.52
INH-014	% Yield	51.35	53.92	2.570	5.004
	% INH released at 4 h in pH 1.2	5.745	8.720	2.975	51.78
	Encapsulation efficiency (%)	90.81	80.77	10.04	11.06
	% INH released at 24 h in pH 6.8	86.62	87.87	1.250	1.443

**Table 9:** ANOVA data for response surface quadratic model (partial sum of squares—Type III) for % RIF released at 0.5 h.

Source	Sum of Squares	Df	Mean Square	F-Value	*p*-Value	Prob > F
**Panel A**						
Model	444.24	9	49.36	75.19	<0.0001	Significant
A-Eudragit^®^ RLPO	38.36	1	38.36	58.43	0.0001	Significant
B-Ethylcellulose	334.76	1	334.76	509.90	<0.0001	Significant
C-d-glucose	8.88	1	8.88	13.52	0.0079	Significant
AB	2.34	1	2.34	3.56	0.1011	-
AC	0.078	1	0.078	0.12	0.7399	-
BC	0.80	1	0.80	1.22	0.3053	-
A^2^	5.24	1	5.24	7.98	0.0256	Significant
B^2^	53.64	1	53.64	81.70	<0.0001	Significant
C^2^	2.57	1	2.57	3.92	0.0881	-
Residual	4.60	7	0.66	-	-	-
Lack of fit	1.79	1	1.79	0.89	0.7200	-
Pure error	0.46	4	0.12	-	-	-
Cor total	448.84	16	-	-	-	-
**Panel B**						
Std. deviation	0.81	-	-	-	-	-
Mean	8.24	-	-	-	-	-
% C.V	9.83	-	-	-	-	-
Press	66.87	-	-	-	-	-
R^2^	0.9898	-	-	-	-	-
Adjusted R^2^	0.9766	-	-	-	-	-
Predicted R^2^	0.8510	-	-	-	-	-
Adeq precision	27.866	-	-	-	-	-

**Table 10:** Summary of best fit models, significant factors, and equations describing the relationship between input variables and response for RIF microspheres.

Response	Fitting Model	Significant Factors	Equation
% RIF released at 0.5 h = R_1_	Quadratic	A-Eudragit^®^ RLPO B-Ethylcellulose C-d-glucose	R_1_ = 7.46 − 2.19 − 6.47B + 1.05C + 0.76AB − 0.14AC + 0.45BC − 1.12A^2^ + 3.57B^2^ − 0.78C^2^
% RIF released at 2 h = R_2_	Quadratic	A-Eudragit^®^ RLPO B-Ethylcellulose	R_2_ =34.44 − 6.59A − 6.42B + 2.42 C + 4.93AB − 1.14 AC + 1.55BC − 6.00A^2^ − 7.86B^2^ − 4.37C^2^
% RIF released at 4 h = R_3_	Quadratic	B-Ethylcellulose C-d-glucose	R_3_ = 47.3 − 0.54A − 8.47B + 2.03 C + 0.41AB − 0.53AC + 2.06BC − 3.94A^2^ − 2.29B^2^ − 4.65C^2^
% RIF released at 6 h = R_4_	Linear	B-Ethylcellulose C-d-glucose	R_4_ = 67.00 − 2.15A − 7.44B + 3.32C
% RIF released at 8 h = R_5_	Quadratic	A-Eudragit^®^ RLPO B-Ethylcellulose C-d-glucose	R_5_ = 83.95 − 3.85A - 8.78B + 2.28C − 0.76AB + 1.12AC + 0.038BC − 3.01A^2^ − 4.23B^2^ − 1.92C^2^
Encapsulation efficiency (%) = R_6_	Quadratic	A-Eudragit^®^ RLPO B-Ethylcellulose C-d-glucose	R_6_ = 77.46 + 9.44A + 4.29B − 1.26C − 2.51AB + 1.37AC + 0.32BC − 0.59A^2^ + 2.14B^2^ + 4.77C^2^
Buoyancy = R_7_	Quadratic	A-Eudragit^®^ RLPO B-Ethylcellulose	R_7_ = 60.17 − 2.60A + 5.10B + 2.07C
Lag time (s) = R_8_	Quadratic	A-Eudragit^®^ RLPO B-Ethylcellulose C-d-glucose	R_8_ =151.00 + 52.88A − 19.75B + 13.13C − 39.25AB − 2.50AC + 13.75BC − 19.00A^2^ − 30.25B^2^ − 14.50C^2^
% RIF released at 12 h = R_9_	Quadratic	A-Eudragit^®^ RLPO B-Ethylcellulose C-d-glucose	R_9_ = 90.37 − 2.12A − 7.80B + 3.04 C + 0.17AB + 1.54 AC + 1.61BC − 4.14A^2^ + 0.74B^2^ − 1.29C^2^
% Yield = R_10_	Quadratic	A-Eudragit^®^ RLPO B-Ethylcellulose	R_10_ = 71.16 + 8.60A + 5.34B − 0.90C − 0.80AB − 0.65AC + 1.04BC + 2.23A^2^ − 2.07B^2^ + 1.93C^2^

**Table 11:** Prediction accuracy for the Box Behnken design optimization process.

Batch	Response	Prediction Accuracy			
		Predicted	Actual	Residual	% P.E.
**A**	% RIF released at 0.5 h (R_1_)	15.39	15.65	0.26	1.66
	% RIF released at 2 h (R_2_)	36.99	38.04	1.05	2.76
	% RIF released at 4 h (R_3_)	48.58	49.18	0.6	1.22
	% RIF released at 6 h (R_4_)	68.71	65.70	3.01	4.58
	% RIF released at 8 h (R_5_)	76.55	78.55	2	2.55
	Encapsulation efficiency (%) (R_6_)	94.71	94.80	0.09	0.095
	% Buoyancy (R_7_)	62.23	58.62	3.61	6.16
	Floating lag time (seconds) (R_8_)	149.63	144	5.63	3.91
	% RIF released at 12 h (R_9_)	92.12	87.89	4.23	4.81
	% Yield (R_10_)	82.19	81.95	0.24	0.29
**B**	% RIF released at 0.5 h (R_1_)	15.39	15.81	0.42	2.66
	% RIF released at 2 h (R_2_)	36.99	38.58	1.59	4.12
	% RIF released at 4 h (R_3_)	48.58	49.11	0.53	1.08
	% RIF released at 6 h (R_4_)	68.71	64.57	4.14	6.41
	% RIF released at 8 h (R_5_)	76.55	77.08	0.53	0.688
	Encapsulation efficiency (%) (R_6_)	94.71	99.19	4.48	4.52
	% Buoyancy (R_7_)	62.23	56.22	6.01	10.69
	Floating lag time (seconds) (R_8_)	149.63	140	9.63	6.88
	% RIF released at 12 h (R_9_)	92.12	86.44	5.68	6.57
	% Yield (R_10_)	82.19	87.97	5.78	6.57
**C**	% RIF released at 0.5 h (R_1_)	15.39	16.03	0.64	3.99
	% RIF released at 2 h (R_2_)	36.99	39.32	2.33	5.93
	% RIF released at 4 h (R_3_)	48.58	47.22	1.36	2.88
	% RIF released at 6 h (R_4_)	68.71	66.73	1.98	2.97
	% RIF released at 8 h (R_5_)	76.55	78.44	1.89	2.41
	Encapsulation efficiency (%) (R_6_)	94.71	96.99	2.28	2.35
	% Buoyancy (R_7_)	62.23	55.81	6.42	11.50
	Floating lag time (seconds) (R_8_)	149.63	142	7.63	5.37
	% RIF released at 12 h (R_9_)	92.12	94.71	2.59	2.74
	% Yield (R_10_)	82.19	85.88	3.69	4.20

**Table 12:** Mean particle size and size distribution of INH microspheres following sieve analysis.

Run	Mean Particle Size	Standard Deviation	% RSD	% Mass Retained on Each Sieve			
				315 µm	800 µm	1250 µm	2000 µm
1	745.99	104.26	13.98	-	44.7	55.3	-
2	680.60	367.07	53.93	-	52.4	47.6	-
3	891.28	161.02	18.07	-	0	56.1	43.9
4	615.85	84.20	13.67	-	58.9	41.1	-
5	494.15	113.84	23.04	-	79.6	19.4	-
6	496.82	105.24	21.18	-	59.5	40.5	-
7	515.07	103.39	20.07	-	58.8	41.2	-
8	903.35	197.10	21.82	-	78.7	21.3	
9	659.30	206.35	31.30	44.3	47.2	8.5	-
10	415.76	76.93	18.50	-	100	-	-
11	693.43	154.20	22.24	-	45.3	54.7	-
12	695.22	124.90	17.97				

**Table 13:** Mean particle size and size distribution of RIF microspheres for BBD, optimized, and modified batches.

Batch	Mean Particle Size	Standard Deviation	% RSD	% Mass of Microspheres Retained per Sieve Size (µm)	
				800	1250
RIF001	515.08	209.79	40.72	95.50	4.50
RIF002	489.77	115.63	23.61	97.27	2.73
RIF003	476.59	188.78	39.61	92.54	7.46
RIF004	459.02	145.89	31.78	94.91	5.09
RIF005	540.00	125.39	23.22	97.33	2.67
RIF006	481.42	92.81	19.28	98.42	1.58
RIF007	541.87	108.41	20.01	96.71	3.29
RIF008	423.19	121.86	28.80	97.88	2.12
RIF009	545.96	185.57	33.99	96.33	3.67
RIF010	452.69	88.34	19.51	100	0.00
RIF011	499.55	177.19	35.47	98.11	1.89
RIF012	601.71	167.94	27.91	81.83	18.17
RIF013	620.07	102.67	16.56	72.78	27.22
RIF014	537.71	128.39	23.88	92.64	7.36
RIF015	576.73	95.39	16.54	93.12	6.88
RIF016	485.38	170.33	35.09	54.18	45.82
RIF017	477.58	102.58	21.48	96.67	3.33
RIF018	547.22	108.63	19.85	94.45	5.55
RIF021	526.49	91.88	17.45	96.77	3.23

**Table 14:** Dissolution of RIF in the presence of INH in pH 1.2, 0.1 M HCl.

Product	Initial µg/mL	Final µg/mL	% Recovery ± SD (n = 6)	% Degradation
Rimactane^®^ 150	461.5	444.5	96.3 ± 0.2	3.70
Rimactane^®^ 150 in presence of BE-TABS Isoniazid 100	461.5	333.6	74.4 ± 0.2	21.9
Rimactane^®^ 150 in presence of INH API	461.5	335.8	72.8 ± 0.2	24.4
RIF API	461.5	444.9	96.4 ± 0.2	3.59
RIF API in presence of INH API	461.5	367.3	79.6 ± 0.1	19.1
Rimactane^®^ 150 in presence INH microspheres	461.5	426.6	92.4 ± 0.3	4.05
RIF API in presence of INH microspheres	461.5	436.7	94.6 ± 0.2	3.86
RIF microspheres in presence of INH microspheres	416.9	398.4	95.6.0 ± 0.1	4.44

## Supplementary Materials

The following are available online at https://www.mdpi.com/1999-4923/12/3/234/s1, Figure S1: Typical DSC thermogram for isonicotinylhydrazide (INH) generated at a heating rate of 10 °C/min over the temperature range 30–400 °C. Figure S2: Typical DSC thermogram for a 1:1 mixture of INH and hydroxypropyl methylcellulose acetate succinate (HPMC-AS) generated at a heating rate of 10 °C/min over the temperature range 30–400 °C. Figure S3: Typical DSC thermogram for a 1:1 mixture of INH and Eudragit® L100 generated at a heating rate of 10 °C/min. over the temperature range 30–400 °C. Figure S4: FT Raman spectral data for INH alone and in microspheres. Figure S5: Typical DSC thermogram for rifampicin (RIF) generated at a heating rate of 10 °C/min over the temperature range 30–400 °C. Figure S6: Typical DSC thermogram for a 1:1 mixture of RIF and d-glucose generated at a heating rate of 10 °C/min over the temperature range 30–400 °C. Figure S7: Typical DSC thermogram for a 1:1 mixture of RIF and EC generated at a heating rate of 10 °C/min over the temperature range 30–400 °C. Figure S8: Typical DSC thermogram for a 1:1 mixture of RIF and Eudragit® RLPO generated at a heating rate of 10 °C/min over the temperature range 30–400 °C. Figure S9: Typical DSC thermogram for a 1:1 mixture of RIF and sodium bicarbonate generated at a heating rate of 10 °C/min over the temperature range 30–400°C. Figure S10: Typical DSC thermogram for a 1:1 mixture of RIF and sodium bicarbonate generated at a heating rate of 10 °C/min over the temperature range 30–400 °C. Figure S11: FTIR absorption spectrum of RIF (black) and a 1:1 mixture of RIF and EC (red). Figure S12: FTIR absorption spectrum of RIF (black) and a 1:1 mixture of RIF and d-glucose (red). Figure S13: FTIR absorption spectrum of RIF (black) and a 1:1 mixture of RIF and Eudragit® RLPO (red). Figure S14: FTIR absorption spectrum of RIF (black) and a 1:1 mixture of RIF and sodium bicarbonate (red). Figure S15: FTIR absorption spectrum of RIF (black) and a 1:1 mixture of RIF and citric acid (red). Table S1: FT Raman shifts and assignments for isonicotinylhydrazide (INH) powder and microspheres. Table S2: FTIR wavenumber number assignments for RIF and 1:1 mixtures. Table S3: Actual experiments conducted and responses observed for RIF using a Box Behknen Design (BBD).

## References

[1] Agrawal S., Singh I., Kaur K.J., Bhade S.R., Kaul C.L., Panchagnula R. (2004). Comparative bioavailability of rifampicin, isoniazid and pyrazinamide from a four drug fixed dose combination with separate formulations at the same dose levels. Int. J. Pharm..

[2] Pillai G., Fourie P.B., Padayatchi N., Onyebujoh P.C., Mcllleron H., Smith P.J., Gabriels G.R. (1999). Recent bioequivalence studies on fixed dose combination antituberculosis drug formulations available on the global market. Int. J. Tuberc. Lung Dis..

[3] Coupe A.J., Davis S.S., Wilding I.R. (1991). Variation in gastrointestinal transit of pharmaceutical dosage forms in healthy subjects. Pharm. Res..

[4] Shishoo C.J., Shah S.A., Rathod I.S., Savale S.S., Vora M.J. (2001). Impaired bioavailability of rifampicin in presence of isoniazid from fixed dose combination (FDC) formulation. Int. J. Pharm..

[5] Sankar R., Sharda N., Singh S. (2003). Behaviour of decomposition of rifampicin in the presence of isoniazid on the pH range 1-3. Drug Dev. Ind. Pharm..

[6] Shishoo C.J., Shah S.A., Rathod I.S., Savale S.S., Kotecha J.S., Shah P.B. (1999). Stability of rifampicin in dissolution medium in presence of isoniazid. Int. J. Pharm..

[7] Singh S., Mariappan T.T., Sharda N., Chakraborti A.K. (2000). The reason for an increase in decomposition of rifampicin in the presence of isoniazid under acid conditions. Pharm. Pharm. Comm..

[8] Maggi N., Pasqualucci C.R., Ballota R., Sensi P. (1966). Rifampicin, a new orally active rifamycin. Chemotherapia.

[9] Sensi P., Maggi N., Furesz S., Maffi G. (1966). Chemical modifications and biological properties of rifamycins. Antimicrob. Agents Chemother..

[10] Singh S., Mariappan T.T., Shankar R., Sarda N., Singh B. (2001). A critical review of the probable reasons for the poor variable bioavailability of rifampicin from anti-tubercular fixed-dose combination (FDC) products, and the likely solutions to the problem. Int. J. Pharm..

[11] Mariappan T.T., Singh S. (2003). Regional gastrointestinal permeability of rifampicin and isoniazid (alone and their combination) in the rat. Int. J. Tuberc. Lung Dis..

[12] Bhise S.B., More A.B., Malayandi R. (2010). Formulation and In Vitro Evaluation of Rifampicin Loaded Porous Microspheres. Sci. Pharm..

[13] Freire D.F., Aragao S.C.F. (2009). Thermal Studies of Isoniazid and Mixtures with Rifampicin. J. Therm. Anal. Calorim..

[14] Gohel C.M., Krishnakant G.S. (2007). A Novel Solid Dosage Form of Rifampicin and Isoniazid with Improved Functionality. AAPS Pharmscitech..

[15] Hadassah M., Swarna K.C.H., Prasanthi D., Sai K.I., Vijaya R.J. (2012). Formulation and Evaluation of Floating Bilayered Tablets of Rifampicin, Isoniazid and Pyrazinamide. Int. J. Pharm. Technol..

[16] Krishna T.V., Reddy M.S. (2014). Formulation and Evaluation of Enteric Coated Pellets of Rifampicin and Isoniazid with Improved Rifampicin Stability. Asian J. Pharm. Res..

[17] Kumar T.U., Vasudevan M. (2012). Novel Sustained Release Swellable Bioadhesive Floating Gastroretentive Drug Delivery System of Bilayer Tablets Containing Rifampicin and Isoniazid. Int. J. Pharm. Sci. Lett..

[18] Pund S., Joshi A., Vasu K., Nivsarkar M., Shishoo C. (2011). Gastroretentive delivery of rifampicin: In vitro mucoadhesion and in vivo gamma scintigraphy. Int. J. Pharm..

[19] Singh H., Bhadari R., Kaur I.P. (2013). Encapsulation of Rifampicin in Solid Lipid Nanoparticulate System to Limit its Degradation and Interaction with Isoniazid at Acidic pH. Int. J. Pharm..

[20] Sullad A.G., Manjeshwar L.S., Aminabhavi T.M. (2010). Novel pH-Sensitive Hydrogels Prepared from the Blends of (Poly(vinyl alcohol) with Acrylic Acid-graft-Guar Gum Matrixes for Isoniazid Delivery. Ind. Eng. Chem. Res..

[21] Khamanga S.M., Parfitt N., Nyamuhiwa T., Haidula H., Walker R.B. (2009). The Evaluation of Eudragit microcapsules manufactured by solvent evaporation using USP Apparatus 1. Dissolut. Technol..

[22] Hwisa N.T., Katakam P., Chandu B.R., Adiki S.K. (2013). Solvent Evaporat ion Techniques as Promising Advancement in Microencapsulation. Vedic Res. Int. Biol. Med. Chem..

[23] Prakash K., Raju P.N., Shanta K.K., Lakshmi M.N. (2007). Preparation and characterization of lamivudine microcapsules using various cellulose polymers. Trop. J. Pharm Res..

[24] Mateovic T., Kriznar B., Bogataj M., Mrhar A. (2002). The influence of stirring rate on biopharmaceutical properties of Eudragit RS microspheres. J. Microencapsul..

[25] Noordin M.Y., Venkatesh V.C., Sharif S., Elting S., Abdullah A. (2004). Application of response surface methodology in describing the performance of coated carbide tools when turning AISI 1045 steel. J. Mater. Process. Technol..

[26] Hill W.J., Hunter W.G. (1966). A Review of response surface methodology, a literature survey. Technometrics.

[27] Kirkwood T.B.L. (1979). Geometric means and measures of dispersion. Biometrics.

[28] Hendricks. W.A., Robey K.W. (1936). The Sampling Distribution of the Coefficient of Variation. Ann. Math. Stat..

[29] Barmpalexis P., Kanaze F.I., Georgarakis E. (2009). Developing and optimising a validated iscocratic reversed-phase high-performance liquid chromatography separation of nimodipine and impurities in tablets using experimental design methodology. J. Pharm. Biomed. Anal..

[30] Box G.E.P., Wilson K.B. (1951). On the Experimental Attainment of Optimum Multifactorial Conditions. R. Stat. Soc..

[31] Carr R.L. (1965). Classifying flow properties of solids. Chem. Eng..

[32] Hausner H.H. (1967). Friction conditions in a mass of metal powders. Int. J. Powder Metall..

[33] Zhang Z., Feng S. (2006). The drug encapsulation efficiency, in vitro release, cellular uptake and cytotoxicity of paclitaxel-loaded poly(lactide)-tocopheryl polyethylene glycol succinate nanoparticles. Biomaterials.

[34] Mwila C., Khamanga S.M.M., Walker R.B. (2016). Development and Assessment of a USP Apparatus 3 Dissolution Test Method for Sustained-Release Nevirapine Matrix Tablets. Dissol Technol..

[35] Ozdemir N., Ordu S., Ozkan Y. (2000). Studies of floating dosage forms of furosemide, in vitro and in vivo evaluations of bilayer tablet formulations. Drug Dev. Ind. Pharm..

[36] Harikumar S.L., Sharma A. (2012). Development and evaluation of bromhexine hydrochloride floating microparticulates. Asian J. Pharm..

[37] Gunasekaran S., Sailatha E., Seshadri S., Kumaresan S. (2009). FTIR, FT Raman Spectra and molecular structural confirmation of isoniazid. Indian. J. Pure Appl. Phys..

[38] Agrawal S., Ashokraj Y., Bharatam P.V., Pillai O., Panchangnula R. (2004). Solid-state characterisation of rifampicin samples and its biopharmaceutic relevance. Eur. J. Pharm. Sci..

[39] Kenechukwu F.C., Momoh M.A. (2016). Formulation, characterisation and evaluation of the effect of polymer concentration on the release behavior of insulin-loaded Eudragit^®^-entrapped mucoadhesive microspheres. Int. J. Pharm. Investig..

[40] Xiao Z., Liu W., Zhu G., Zhou R., Niu Y. (2014). Production and characterization of multinuclear microcapsules encapsulating lavender oil by complex coacervation. Flavour Fragr. J..

[41] Rowe R.C., Sheskey P.J., Owen S.C. (2006). Polymethacrylates.

[42] Friesen D.T., Shanker R., Crew M., Smithey D.T., Curatolo W.J., Nightingale J.A.S. (2008). Hydroxypropyl Methylcellulose Acetate Succinate-Based Spray-Dried Dispersions: An Overview. Mol. Pharm..

[43] Fu X., Ping Q., Gao Y. (2005). Effects of formulation factors on encapsulation efficiency and release behaviour in vitro of huperzine A-PLGA microspheres. J. Microencapsul..

[44] Li X., Deng X., Yuan M., Xiong C., Huang Z., Zhang Y., Jia W. (1999). Investigation on process parameters involved in preparation of poly-DL-lactide-poly(ethylene glycol) microspheres containing Leptospira Interrogans antigens. Int. J. Pharm..

[45] Mehta R.C., Thanoo B.C., Deluca P.P. (1996). Peptide containing microspheres from low molecular weight and hydrophilic poly(d,l-lactide-co-glycolide). J. Control. Release.

[46] Raval J.P., Naik D.R., Amin K.A., Patel P.S. (2014). Controlled-release and antibacterial studies of doxycycline-loaded poly(å-caprolactone) microspheres. J. Saudi Chem. Soc..

[47] Rafati H., Combes A.G.A., Adler J., Davis S.S. (1997). Protein-loaded poly(DL-lactide-co-glycolide) microparticles for oral administration, Formulation, structural and release characteristics. J. Control. Release.

[48] Bodmier R., McGinity J.W. (1988). Solvent selection in the preparation of PLA microspheres prepared by the solvent evaporation method. Int. J. Pharm..

[49] Wilson B., Babubhai P.P., Sajeev M.S., Jenita J.L., Priyadarshini S.R. (2013). Sustained release enteric coated tablets of pantoprazole: Formulation, in vitro and in vivo evaluation. Acta Pharma.

[50] Kim B.K., Hwang S.J., Park J.B., Park H.J. (2002). Preparation and characterization of drug-loaded polymethacrylate microspheres by an emulsion solvent evaporation method. J. Microencapsul..

[51] Viswanathan N.B., Thomas P.A., Pandit J.K., Kulkarni M.G., Mashelkar R.A. (1999). Preparation of non-porous microspheres with high entrapment efficiency of proteins by a (water-in-oil)-in-oil emulsion technique. J. Control. Release.

[52] Jelvehgari M., Dastmalch S., Nazila D. (2012). Theophylline-ethylcellulose microparticles, screening of the process and formulation variables for preparation of sustained release particles. Iran. J. Med. Sci..

[53] Jelvehgari M., Montazam S.H. (2012). Comparison of microencapsulation by emulsion-solvent extraction/evaporation technique using derivatives cellulose and acrylate-methacrylate copolymer as carriers. J. Nat. Pharm. Prod..

[54] Jain S., Srinath M.S., Narendra C., Reddy S.N., Sindhu A. (2010). Development of a floating dosage form of ranitidine hydrochloride by statistical optimisation technique. J. Young Pharm..

[55] Kilacarslan M., Baykara T. (2003). The effect of drug polymer ratio on the properties of the verapamil HCl loaded microspheres. Int. J. Pharm..

[56] Chandran S., Asghar L.F.A., Mantha N. (2008). Design and evaluation of ethyl cellulose based matrix tablets of ibuprofen with pH modulated release kinetics. Ind J. Pharm. Sci..

[57] Ozturk A.G., Ozturk S.S., Palsson B.O., Wheatley T.A., Dressman J.B. (1990). Mechanism of release from pellets coated with an ethylcellulose-based film. J. Control. Release..

[58] Bodmeir R., Paeratakul O. (1994). The effect of curing on drug release and morphological properties of ethylcellulose pseudolatex-coated beads. Drug Dev. Ind. Pharm..

[59] Khan G.M., Meidan V.M. (2007). Drug release kinetics from tablet matrices based upon ethylcellulose ether-derivatives, a comparison between different formulations. Drug Dev. Ind. Pharm..

[60] Sengel-Turk C.T., Hascicek C., Gonul N. (2011). Ethylcellulose-based matrix-type microspheres, influence of plasticizer ratio as pore-forming agent. AAPS Pharmscitech..

[61] Schnieders J., Gbureck U., Vorndran E., Schossik M., Kissel T. (2011). The effect of porosity on drug release kinetics from vancomycin microsphere/calcium phosphate cement composites. J. Mater. Res..

[62] Emeje M.O., Kunle O.O., Ofoefule S.I. (2006). Compaction characteristics of ethylcellulose in the presence of some chanelling agents, technical note. AAPS Pharmscitech..

[63] Zhi-wei Y., Patrick R., Jean P.R., Chris V. (2007). Correlation between permeability of metoprolol tartrate through plasticized isolated ethylcellulose/hydroxypropyl methylcellulose films and drug release from reservoir pellets. Eur. J. Pharm. Biopharm..

[64] Rowe R.C. (1986). The effect of the molecular weight of ethyl cellulose on the drug release properties of mixed films of ethyl cellulose and hydroxypropylmethylcellulose. Int. J. Pharm..

[65] Murtasa G. (2012). Ethylcellulose microparticles, a review. Acta Pol. Pharm.-Drug Res..

[66] Katayoun D., Marzieh S. (2010). Formulation and in vitro evaluation of nifedipine controlled release tablet: Influence of combination of hydrophylic and hydrophobic matrix forms. Asian J. Pharm..

[67] Kang F., Singh J. (2001). Effect of additives on the release of a model protein from PLGA microspheres. AAPS PharmSciTech.

[68] Patra C.N., Kumar A.B., Pandit H.K., Singh S.P., Devi M.V. (2007). Design and evaluation of sustained release bilayer tablets of propranolol hydrochloride. Acta Pharm..

[69] Khandai M., Chakraborty S., Sharma A., Panda D., Khanam N., Panda S.K. (2010). Development of propranolol hydrochloride matrix tablets, an investigation on effects of combination of hydrophilic and hydrophobic matrix formers using multiple comparison analysis. Int. J. Pharm. Sci. Rev. Res..

[70] Jagtap Y.M., Bhujbal R.K., Ranade A.N., Ranpise N.S. (2012). Effect of various polymers concentrations on physicochemical properties of floating microspheres. Ind. J. Pharm. Sci..

[71] Pande A.V., Vaidya P.D., Arora A., Dhoka M.V. (2010). In vitro and in vivo evaluation of ethyl cellulose based floating microspheres of cefpodoxime proxetil. Int. J. Biomed. Res..

[72] de la Cruz G.V., Torres J.A., Martin-Polo M.O. (2001). Temperature effect on the moisture sorption isotherms for methylcellulose and ethylcellulose films. J. Food Eng..

[73] Saravanan M., Anupama B. (2011). Development and evaluation of ethylcellulose floating microspheres loaded with ranitidine hydrochloride by novel solvent evaporation-matrix erosion method. Carbohyd. Polym..

[74] Pandya N., Pandya M., Bhaskar V.H. (2011). Preparation and in vitro characterisation of porous carrier–based glipizide floating microspheres for gastric delivery. J. Young Pharm..

[75] Amin L., Ahmed T., Mannan A. (2016). Development of floating-mucoadhesive microsphere for site specific release of metronidazole. Adv. Pharm. Bull..

[76] Akbuga J. (1989). Preparation and evaluation of controlled release furosemide microspheres by spherical crystallisation. Int. J. Pharm..

[77] Chemtob C., Chaumeil J.C., N’Dongo M. (1986). Microencapsulation by ethylcellulose phase separation: Microcapsule characteristics. Int. J. Pharm..

[78] Huang X., Cheng X., Luo Y., Guo Z., Zhong C., Zhang Y. (2015). Effect of homogenisation speed on morphology and release of protein loaded PLGA microspheres made by W/O/W method. Acta Sci. Nat. Univ. Sunyatseni.

[79] Kim K.K., Pack D.W., Ferrari M., Lee A.P., Lee L.J. (2006). Microspheres for Drug delivery. BioMEMS and Biomedical Nanotechnology.

[80] Stevens R.E., Dorantes A., Gray V., Pham L. (2015). Scientific and Regulatory Standards for Assessing Product Performance Using the Similarity Factor, f2. AAPS J..

[81] Prasanthi B., D.Prasanthi D., Bhavani B., Ratna J.V. (2014). A novel drug delivery system designed for modulating the release kinetics of antitubercular drugs. Int. J. Pharm..

[82] Acocella G. (1978). Clinical pharmacokinetics of rifampicin. Clin. Pharm..

