# Supplementary material for: Improved Stability of Rifampicin in the Presence of Gastric-Resistant Isoniazid Microspheres in Acidic Media
Source: Pharmaceutics. 2020 Mar 5;12(3):234. doi: 10.3390/pharmaceutics12030234 (PMC7150845; doi:10.3390/pharmaceutics12030234)
Supplement: Supplementary file 1 [file pharmaceutics-12-00234-s001.pdf]

pharmaceutics

Supplementary Materials: Improved Stability of Rifampicin in the Presence of Gastric-Resistant Isoniazid Microspheres in Acidic Media

Chiluba Mwila and Roderick B. Walker

1. Isoniazid (INH)-Excipient Compatibility

1.1. Differential Scanning Calorimetry

The DSC thermograms for isoniazid (INH), 1:1 mixtures of INH with hydroxypropyl methylcellulose acetate succinate (HPMC-AS) or Eudragit® L100 are depicted in Figures S1–S3.

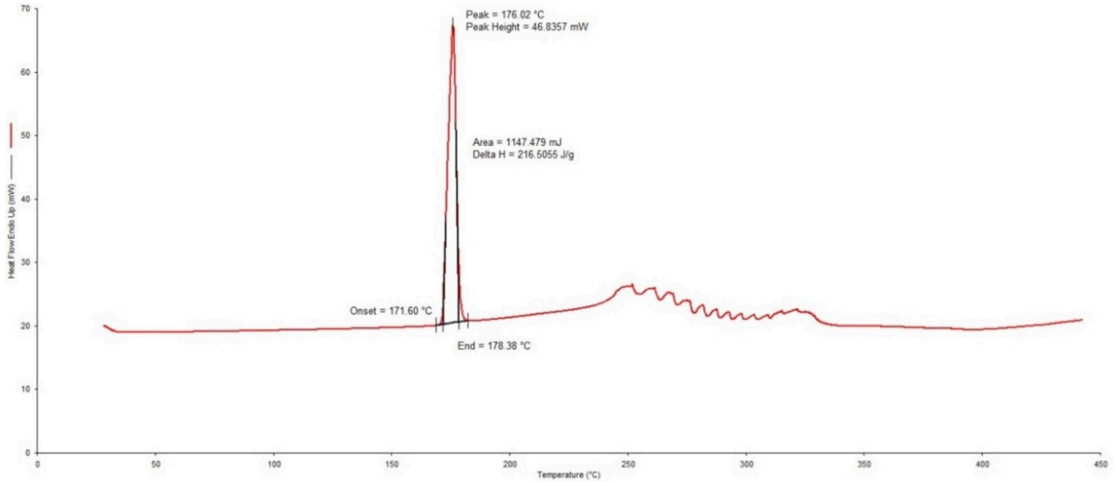

Figure S1. Typical DSC thermogram for isoniazid (INH) generated at a heating rate of 10 °C/min over the temperature range 30–400 °C.

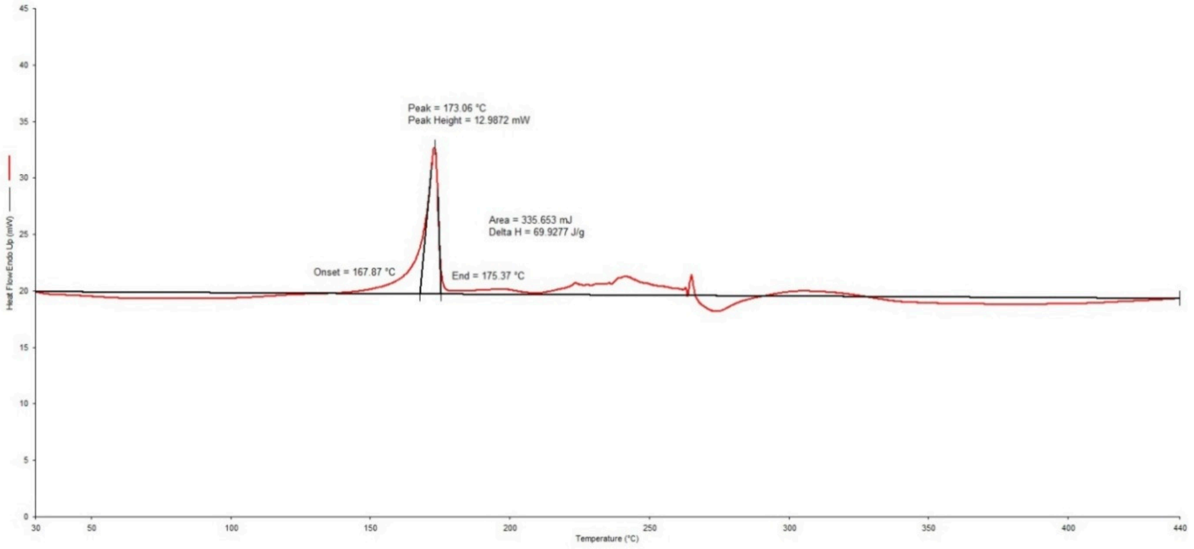

Figure S2. Typical DSC thermogram for a 1:1 mixture of isoniazid (INH) and hydroxypropyl methylcellulose acetate succinate (HPMC-AS) generated at a heating rate of 10 °C/min over the temperature range 30–400 °C.

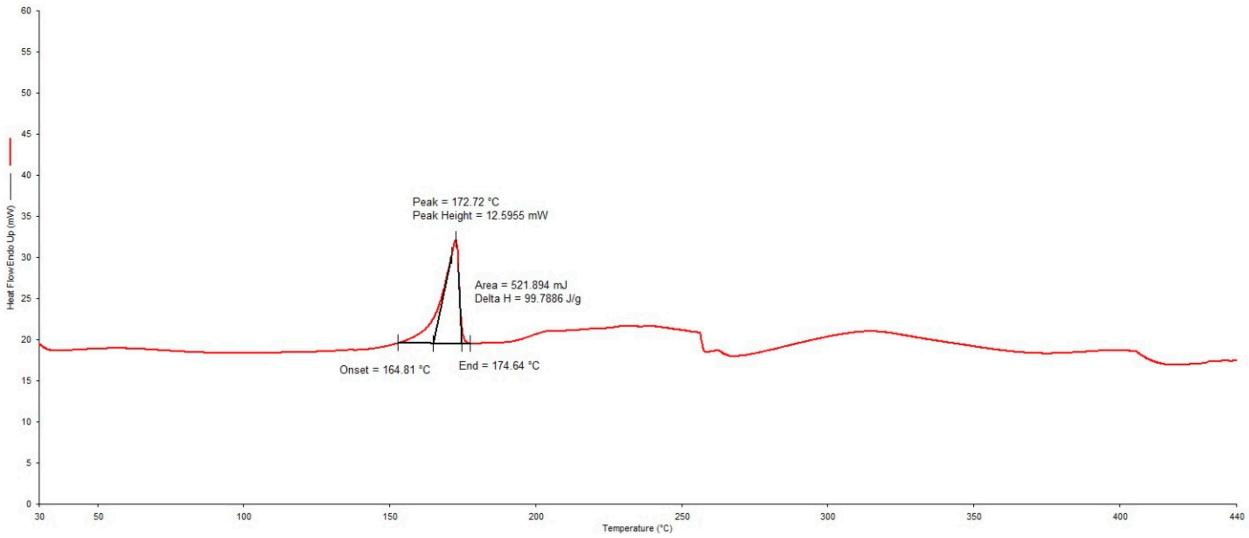

Figure S3. Typical DSC thermogram for a 1:1 mixture of isoniazid (INH) and Eudragit® L100 generated at a heating rate of 10 °C/min. over the temperature range 30–400 °C.

The DSC thermogram for INH revealed the presence of a single sharp endothermic peak between 171.60–178.38 °C with the apex at 176.02 °C corresponding to the melting point of INH [1]. The sharp peak confirms the crystalline nature of INH and the background noise at 250 °C corresponding to the degradation of INH. The thermogram of a 1:1 mixture of INH and HPMC-AS revealed the presence of an endothermic peak for INH between 167.87 °C and 175.37 °C with an apex at 173.06 °C and the thermogram for the 1:1 mixture of INH and Eudragit® L100 revealed the presence of an endothermic peak for INH between 164.81 °C and 174.64 °C with the apex at 172.72 °C. The thermograms of the mixtures of INH and polymers revealed a minimal decrease in the melting point of INH with slight broadening at the base of the peak, indicating a slight reduction in the crystallinity of INH. These results do not however, suggest a potential or significant incompatibility between INH and the excipient tested, However, real-time long-term stability studies would be required to ensure that this is indeed the case.

1.2. FT Raman Spectroscopy

The FT Raman spectra for INH alone and in microspheres in the presence of all excipients are depicted in Figure S4. The comparative frequencies for important functional groups of INH and the microspheres, in relation to what has been reported [2] are summarised in Table S1.

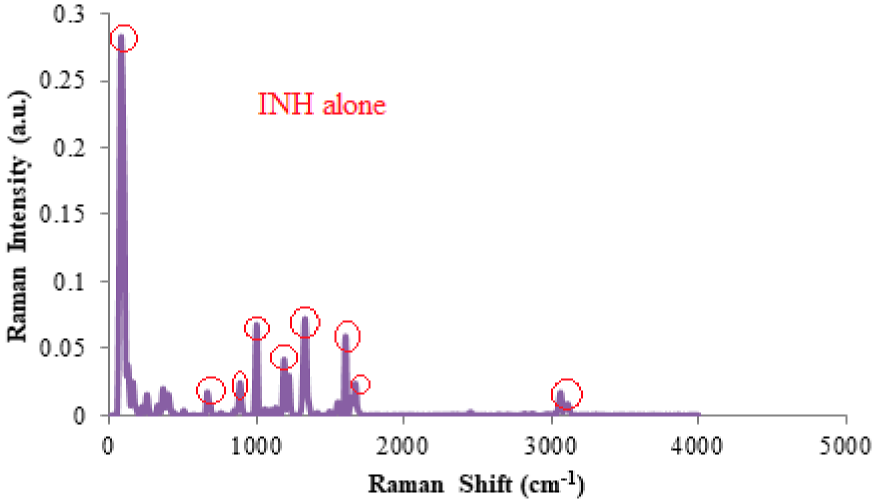

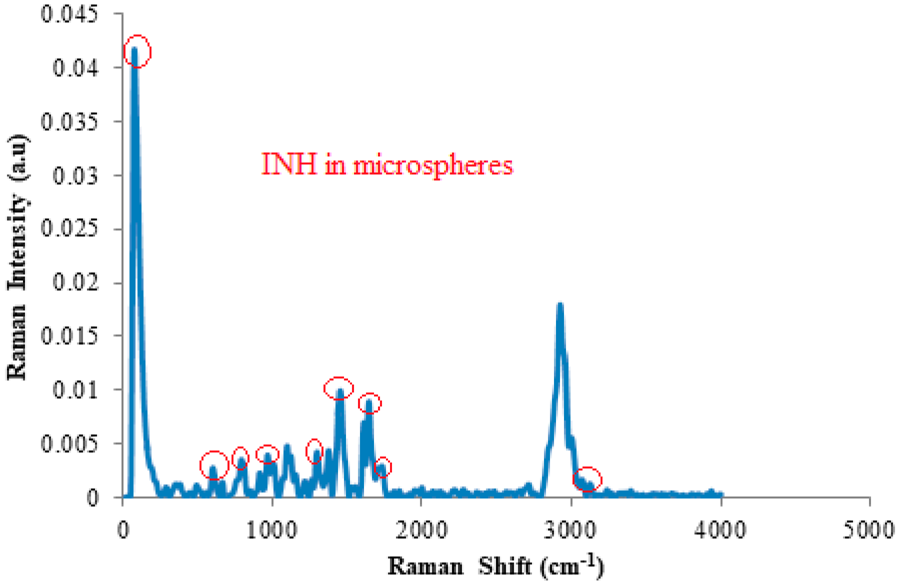

Figure S4. FT Raman spectral data for INH alone and in microspheres.

Table S1. FT Raman shifts and assignments for isonizaid (INH) powder and microspheres.

Experimental Wavenumber (cm^{-1})		Literature Wavenumber (cm^{-1})	Functional Group Assignment
INH	Microspheres		
1410.054	1410.054	1410	Ring C=C symmetrical stretching
1550.832	1550.832	1551	Ring C=N symmetrical stretching
1057.146	1057.146	1057	C–C stretching
1668.467	1668.467	1669	C=O stretching
1330.987	1330.987	1331	C–N stretching
3300.132	3301.872	3300	N–H stretching
1130.428	1130.428	1131	N–NH_2 stretching
503.6787	503.6787	504	Ring C–C–C symmetrical bending
848.8727	848.8727	849	Ring C–C–H symmetric bending
665.6692	665.6692	666	–C–C–C bending
681.0968	681.0968	682	–C–C=O bending
1552.76	1552.76	1551	H–N–N bending
1494.906	1494.906	1495	C–N–H bending
1602.9	1602.9	1602	Ring C=C asymmetrical stretching
1641.469	1641.469	1642	Ring C=N Asymmetric stretching

2979.919	2979.819	2980	C–H asymmetrical stretching
3064.671	3064.671	3065	C–H asymmetrical stretching
750.5213	750.5213	750	Ring C–C–C asymmetric bending
1001.221	1001.221	1002	Ring C–C–N asymmetric bending
1186.353	1186.353	1187	Ring C–C–H asymmetric bending
1219.137	1219.137	1219	Ring C–C–H asymmetric bending
887.7419	887.4419	887	Ring C–N–C bending

The FT Raman spectroscopic study of the microspheres confirmed the presence of all relevant functional groups for INH and the absence of potential detrimental interactions between INH when incorporated into a microsphere formulation in combination with HPMC-AS and Eudragit® L100. However, real-time long-term stability studies would be required to ensure that this is indeed the case.

2. Rifampicin (RIF)-Excipient Compatibility Studies

2.1. Differential Scanning Calorimetry

The DSC thermograms for rifampicin (RIF) is depicted in Figure S5.

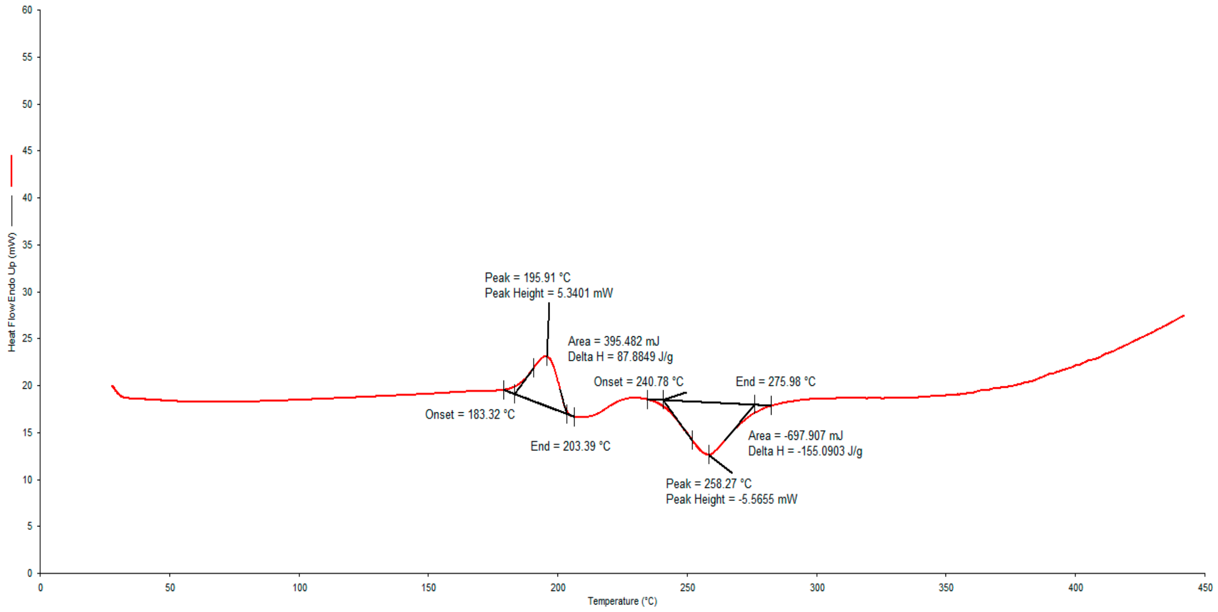

Figure S5. Typical DSC thermogram for rifampicin (RIF) generated at a heating rate of 10 °C/min over the temperature range 30–400 °C.

The DSC thermogram for RIF was characterized by a melting endotherm between 183.32–203.39 °C, immediately followed by an exothermic recrystallisation event that is a characteristic of a solid-liquid-solid transition, and finally a decomposition event at 240.78–275.58 °C. These results were similar to those in which the form II polymorph of RIF exhibited a melting endotherm at between 180–197 °C that was immediately followed by recrystallisation to the form I polymorph with an exothermic peak at between 197–223 °C and a final decomposition event that was observed between 247–266 °C [3].

The DSC thermogram for a 1:1 mixture of RIF and d-glucose is depicted in Figure S6.

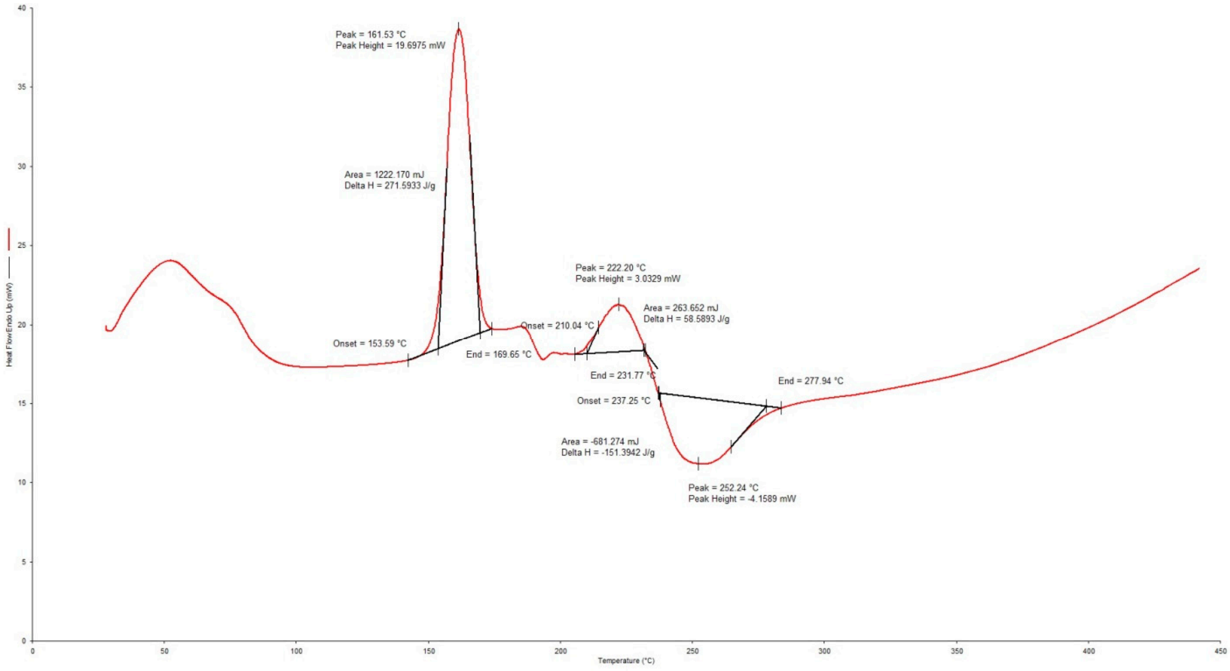

Figure S6. Typical DSC thermogram for a 1:1 mixture of rifampicin (RIF) and d-glucose generated at a heating rate of 10 °C/min over the temperature range 30–400 °C.

The thermogram for the 1:1 mixture of RIF and d-glucose revealed a melting endotherm for RIF between 210.04–231.77 °C, no recrystallisation exothermic event and decomposition between 231.77–279.94°C. This thermal behaviour suggested a more rapid transition from polymorphic form II to form I, in the presence of d-glucose, however, this phenomenon has no biopharmaceutical significance but can be attributed to a decrease in melting point of RIF in the presence of d-glucose.

The thermogram for the 1:1 mixture of RIF and ethyl cellulose (EC) revealed a melting endotherm for RIF between 184.02–202.66 °C, immediately followed by a recrystallisation event at 202.66–239.69 °C and decomposition between 239.69–271.92 °C and is depicted in Figure S7.

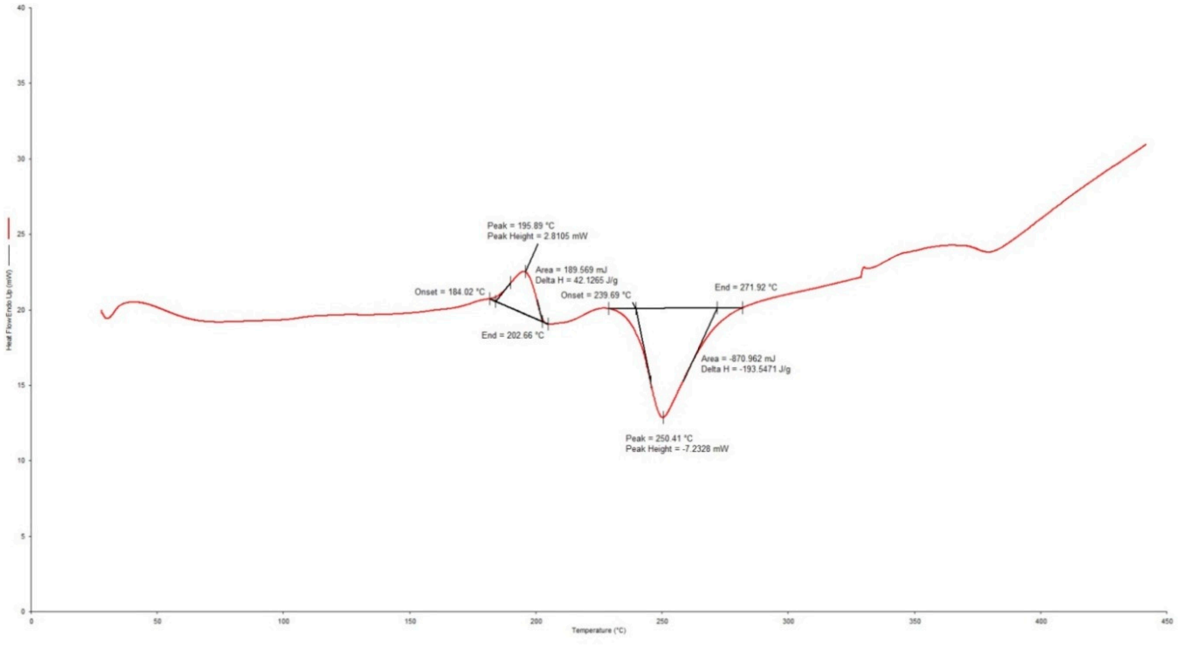

Figure S7. Typical DSC thermogram for a 1:1 mixture of rifampicin (RIF) and ethyl cellulose (EC) generated at a heating rate of 10 °C/min over the temperature range 30–400 °C.

The thermogram for the 1:1 mixture of RIF and Eudragit® RLPO revealed a melting endotherm for RIF between 182.19–204.04 °C, a recrystallisation event at 204.04–232.18 °C and decomposition between 232.18–276.15 °C and is depicted in Figure S8.

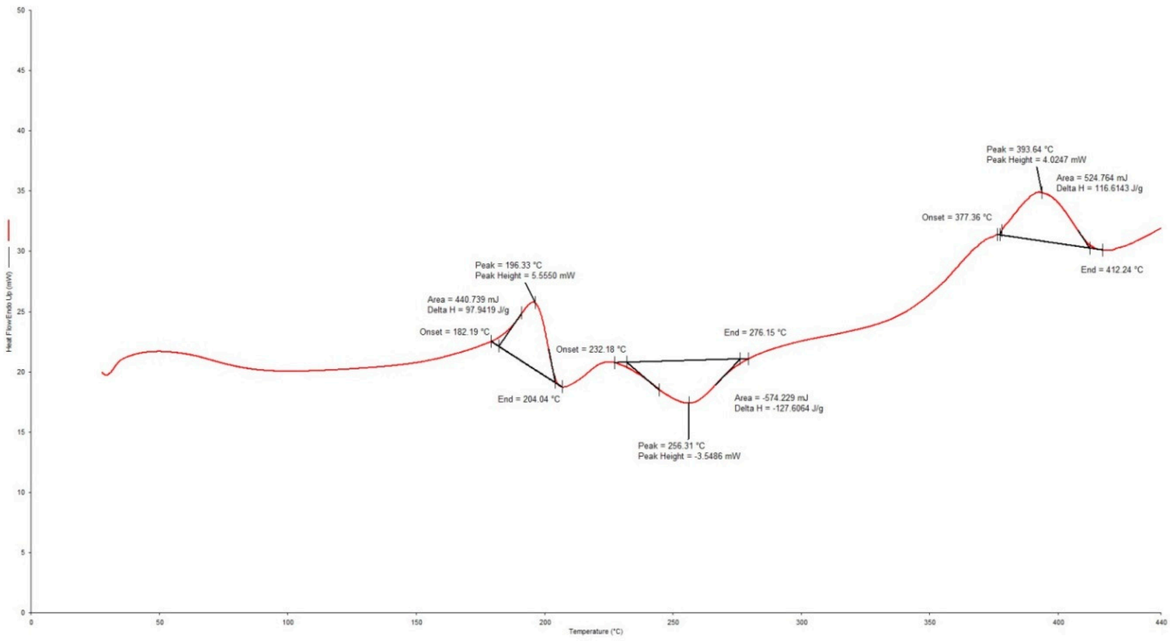

Figure S8. Typical DSC thermogram for a 1:1 mixture of rifampicin (RIF) and Eudragit® RLPO generated at a heating rate of 10 °C/min over the temperature range 30–400 °C.

The thermogram for the 1:1 mixture of RIF and sodium bicarbonate is depicted in Figure S9 and revealed overlapping endothermic peaks at 137.82–194.66 °C which were identified as melting endotherms for sodium bicarbonate and polymorph form II of RIF. The overlapping endothermic peaks were immediately followed by a period of recrystallisation at between 194.66–240.59 °C and decomposition at 240.59–273.46 °C. The thermogram for sodium bicarbonate alone has been reported to depict two endothermic events, one at 96 °C and one at 150 °C that is evidence of thermal decomposition of $NaHCO_3$ generating H_2O and CO_2 and sodium carbonate and commences at approximately 50 °C and ends at 170 °C.

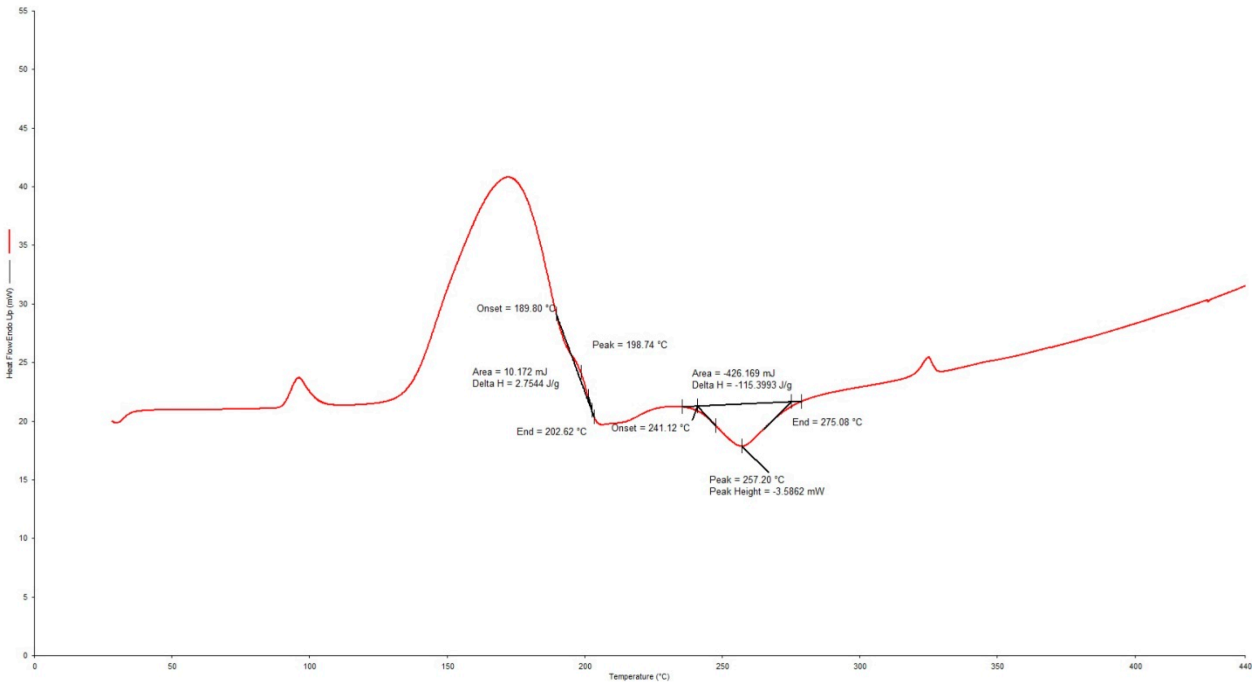

Figure S9. Typical DSC thermogram for a 1:1 mixture of rifampicin (RIF) and sodium bicarbonate generated at a heating rate of 10 °C/min over the temperature range 30–400°C.

The thermogram for the 1:1 mixture of RIF and anhydrous citric acid is depicted in Figure S10 and revealed a melting endotherm for RIF at 175.81–200.49 °C, a recrystallisation event at 200.49 °C and decomposition between 238.41–279.18 °C.

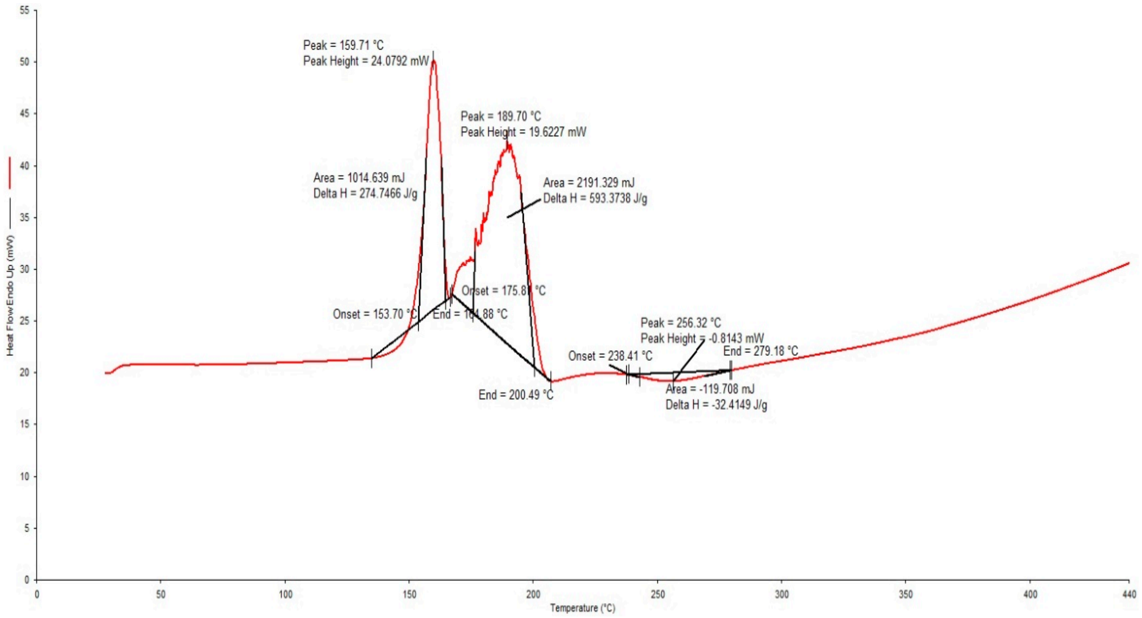

Figure S10. Typical DSC thermogram for a 1:1 mixture of rifampicin (RIF) and sodium bicarbonate generated at a heating rate of 10 °C/min over the temperature range 30–400 °C.

The overlapping peaks in the thermogram for the 1:1 mixture of RIF and sodium bicarbonate, in addition to the interference with the melting endotherm for RIF from possible degradation products of anhydrous citric acid in the thermogram of the 1:1 mixture of RIF and citric acid makes the analysis cumbersome and suggests that other analytical techniques would be more useful in determining the compatibility of RIF with these excipients and that long term stability of the manufactured product

containing RIF, sodium bicarbonate and citric acid should be assessed. These results suggest that the intended excipients were compatible with RIF and may be used in the same formulation composition, however, real-time long-term stability studies would be required to confirm this is indeed, the case.

2.2. FTIR Spectroscopy

The FTIR spectra of 1:1 mixtures of RIF and EC, d-glucose, Eudragit® RLPO, sodium bicarbonate and citric acid are depicted in Figures S11–S15.

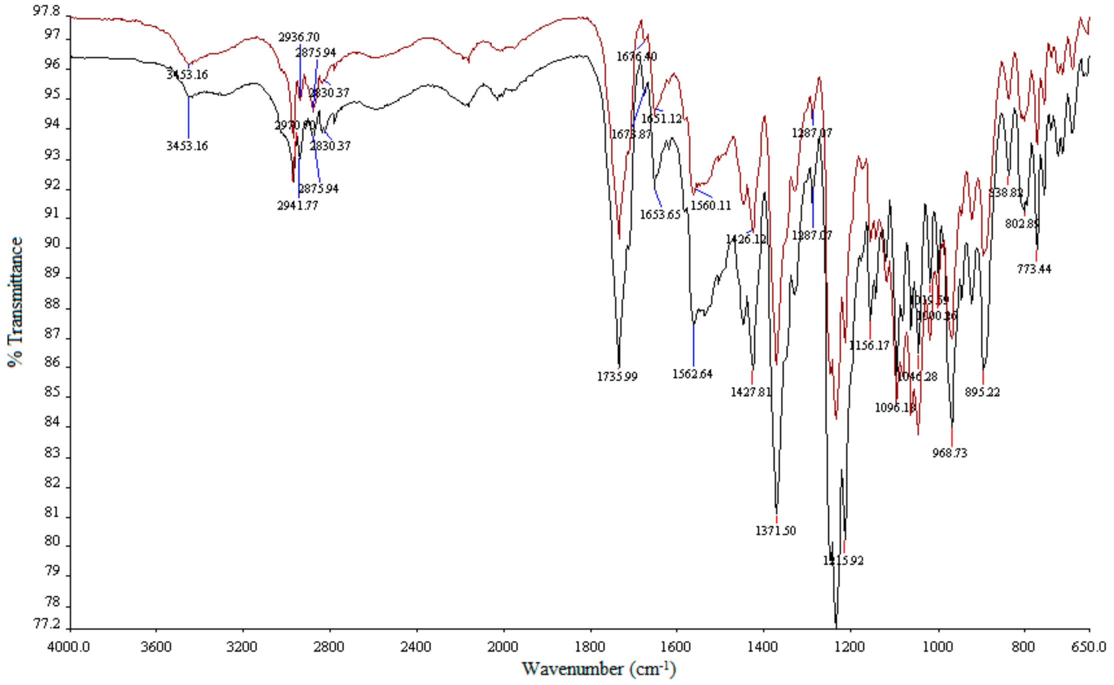

Figure S11. FTIR absorption spectrum of rifampicin (RIF) (black) and a 1:1 mixture of rifampicin (RIF) and ethyl cellulose (EC) (red).

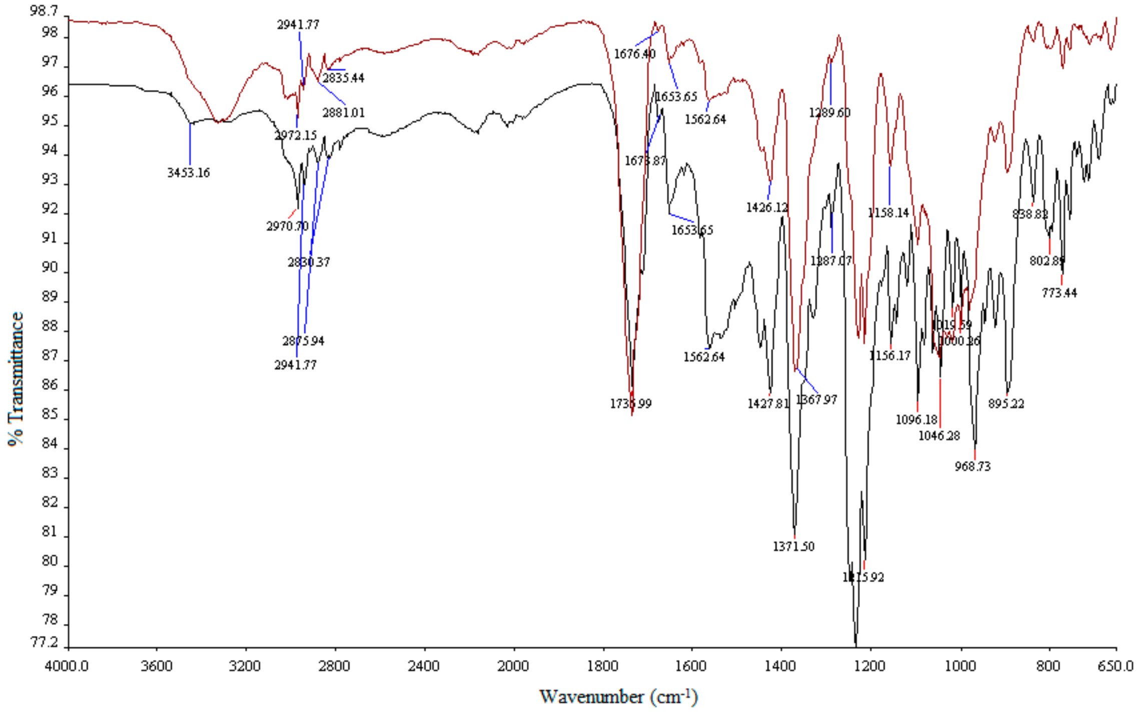

Figure S12. FTIR absorption spectrum of rifampicin (RIF) (black) and a 1:1 mixture of rifampicin (RIF) and d-glucose (red).

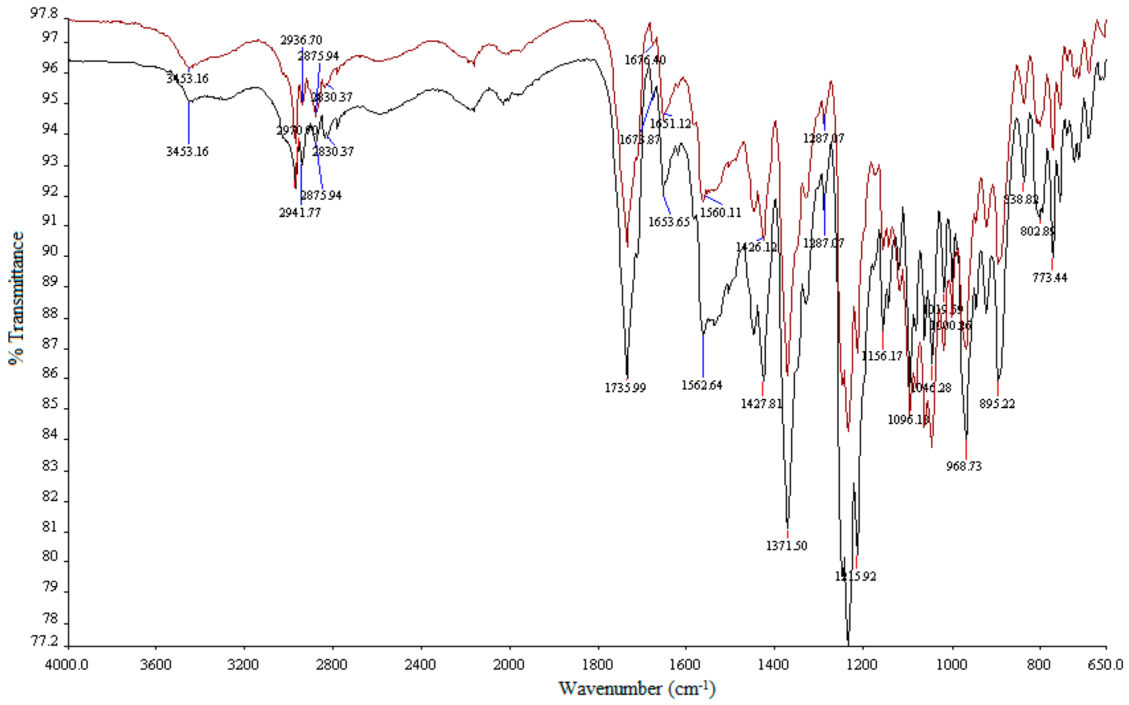

Figure S13. FTIR absorption spectrum of rifampicin (RIF) (black) and a 1:1 mixture of rifampicin (RIF) and Eudragit® RLPO (red).

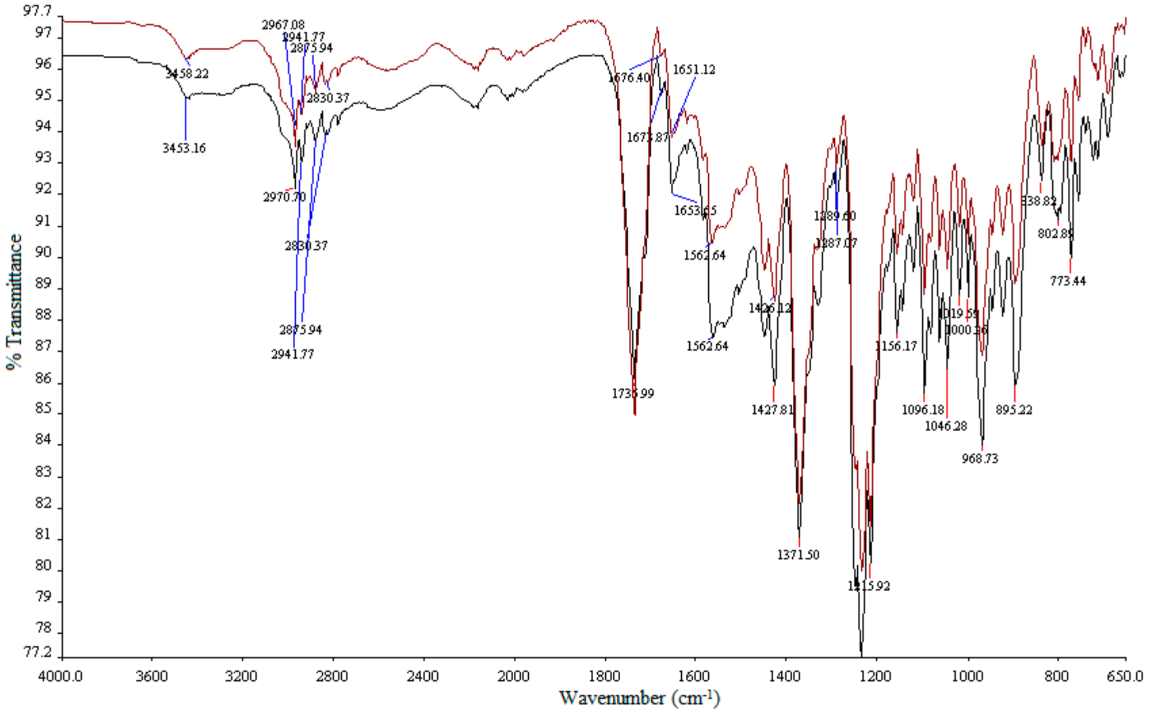

Figure S14. FTIR absorption spectrum of rifampicin (RIF) (black) and a 1:1 mixture of rifampicin (RIF) and sodium bicarbonate (red).

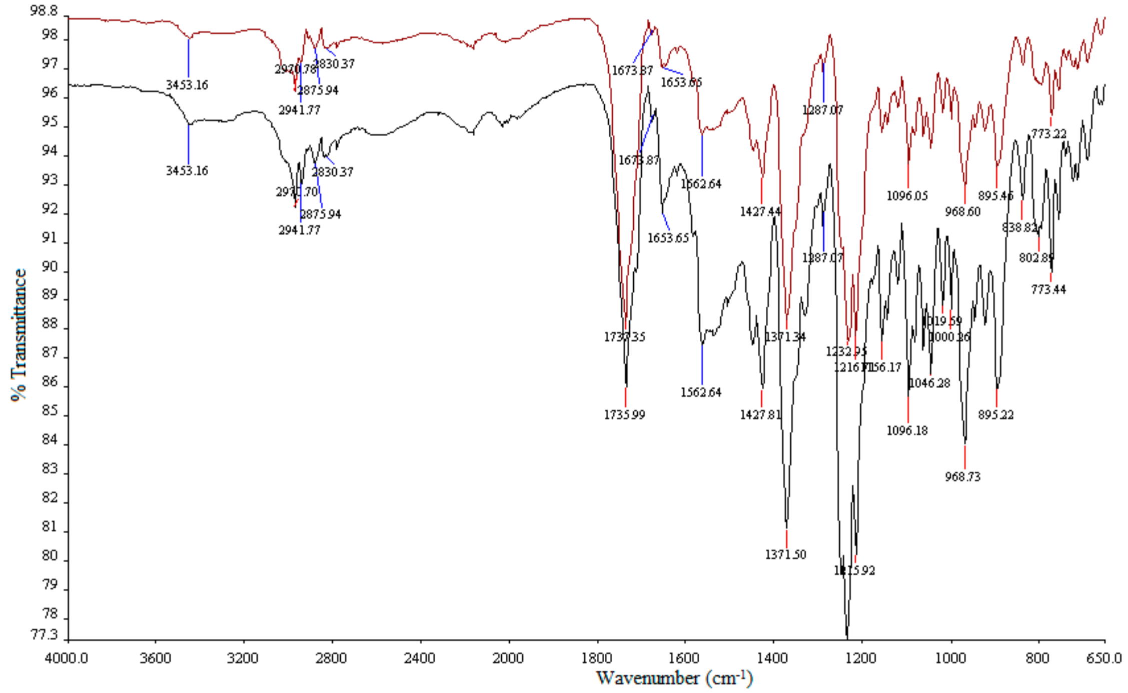

Figure S15. FTIR absorption spectrum of rifampicin (RIF) (black) and a 1:1 mixture of rifampicin (RIF) and citric acid (red).

The infrared absorption spectra for the 1:1 mixtures do not reveal any significant shifts in the frequency of resonation for RIF and the minor shifts in the FTIR frequencies of resonance observed for the functional groups of RIF (Table S2) were attributed to potential weak hydrogen bonding, further confirming the absence of potential interactions between RIF and the excipients to be used for the manufacture of the floating microspheres. However, real-time long-term stability studies would be necessary to ensure this is, indeed the case.

Table S2. FTIR wavenumber number assignments for rifampicin (RIF) and 1:1 mixtures.

Functional Group	RIF	1:1 Mixture of RIF and				
		Ethyl Cellulose	Eudragit® RLPO	d-glucose	Sodium Bicarbonate	Citric Acid
–CH_3 stretching	2941.77	2936.70	2936.70	2941.77	2941.77	2941.78
–CH_3O asymmetric stretching	2875.94	2875.94	2875.94	2881.01	2875.94	2875.94
–C=O acetyl stretching	1735.99	1735.99	1735.99	1735.99	1735.35	1735.99
–C=N asymmetric bending	1673.87	1676.40	1676.40	1676.40	1676.40	1673.87
–C=C stretching	1562.64	1560.11	1560.11	1560.11	1562.40	1562.64
–C–N- stretching	1427.81	1426.10	1426.12	1426.12	1426.12	1427.44

pharmaceutics

MDPI

Table S3. Actual experiments conducted and responses observed for rifampicin (RIF) using a Box Behnken Design (BBD).

Ru (n)	Eudragit® RLPO (g)	Ethyl-cellulose (g)	d-glucose (g)	RIF Released at 0.5 h (%)	RIF Released at 2 h (%)	RIF Released at 4h (%)	RIF Released at 6h (%)	RIF Released at 8h (%)	EE (%)	Buoyancy % ± SD	Lag Time Seconds ± SD	RIF released at 12h (%)	Yield (%)
				R_1	R_2	R_3	R_4	R_5	R_6	R_7	R_8	R_9	R_{10}
1	3	1.5	0.75	3.85	16.94	41.01	68.34	79.86	88.36	56.44 ± 0.22	180 ± 3.6	88.84	81.86
2	3	1	0.5	14.21	19.65	46.10	73.66	81.94	88.23	52.56 ± 0.34	220 ± 2.8	92.18	76.23
3	2	1.5	0.5	7.41	34.40	47.49	69.17	84.39	77.07	59.72 ± 0.08	150 ± 4.9	90.51	70.00
4	1	2	0.5	4.08	11.63	35.24	56.13	72.99	74.80	69.44 ± 0.11	62 ± 6.7	81.43	68.00
5	2	1.5	0.5	7.03	34.06	47.12	69.99	83.37	77.20	58.00 ± 0.72	152 ± 5.4	90.61	71.30
6	3	1.5	0.25	2.61	13.70	39.56	58.29	71.01	90.80	51.76 ± 0.93	170 ± 3.9	79.03	84.22
7	2	1.5	0.5	7.27	34.57	47.44	69.98	84.39	77.26	58.40 ± 0.87	152 ± 4.6	90.02	71.67
8	3	2	0.5	1.53	12.64	30.72	50.64	63.24	91.63	68.52 ± 0.75	80 ± 4.4	75.29	85.33
9	2	1.5	0.5	7.92	34.21	47.38	69.94	83.65	76.78	57.02 ± 1.02	151 ± 4.8	90.42	71.50
10	2	2	0.25	2.61	14.16	26.66	59.08	67.54	88.35	64.60 ± 0.98	65 ± 3.2	78.51	76.56
11	2	1.5	0.5	7.65	34.95	47.06	69.52	83.92	78.98	56.92 ± 2.77	150 ± 4.5	90.28	71.33
12	1	1.5	0.75	8.79	36.70	48.92	70.98	84.78	69.72	65.00 ± 1.87	70 ± 6.8	87.79	67.71
13	1	1.5	0.25	7.00	28.92	35.35	66.54	80.40	77.65	60.44 ± 1.39	50 ± 6.5	84.12	67.47
14	2	1	0.25	15.17	26.08	48.46	70.57	85.54	80.22	58.48 ± 0.79	110 ± 7.3	95.71	68.00
15	2	1	0.75	16.98	27.15	49.95	74.21	87.97	79.74	62.96 ± 2.88	120 ± 28	97.91	63.39
16	1	1	0.5	19.82	38.37	52.23	74.46	88.65	61.37	55.20 ± 1,67	45 ± 4.9	98.99	55.70
17	2	2	0.75	6.21	21.42	36.36	67.52	70.11	89.13	67.40 ± 0.58	130 ± 3.7	87.16	76.11

pharmaceutics

References

1. Pharmacopoeia, B. *British Pharmacopoeia Commission, 2009*; The Stationery Office: London, UK, 2008; Volume I & II ,pp. 3259–3261.
2. Gunasekaran, S.; Sailatha, E.; Seshadri, S.; Kumaresan, S. FTIR, FT Raman Spectra and molecular structural confirmation of isoniazid. *Indian J. Pure Appl. Phys*. **2009**, *47*, 12–18.
3. Agrawal, S.; Ashokraj, Y.; Bharatam, P.V.; Pillai, O.; Panchangnula, R. Solid-state characterisation of rifampicin samples and its biopharmaceutic relevance. *Eur J Pharm Sci*. **2004**, *22*, 127–144.
